# A Dual-Strategy Cuckoo Catfish Optimizer for Continuous Optimization with Application to Wastewater Treatment

**DOI:** 10.3390/biomimetics11100736

**Published:** 2026-10-14

**Authors:** Zongkun Li, Shanfa Tang

**Affiliations:** 1 School of Petroleum Engineering, Yangtze University, Wuhan 430100, China; 2022730021@yangtzeu.edu.cn; 2 Hubei Key Laboratory of Oil and Gas Drilling and Production Engineering, Yangtze University, Wuhan 430100, China; 3 National Engineering Research Center for Oil & Gas Drilling and Completion Technology, Yangtze University, Wuhan 430100, China; 4 State Key Laboratory of Low Carbon Catalysis and Carbon Dioxide Utilization, Wuhan 430100, China

**Keywords:** cuckoo catfish optimizer, crisscross search, Cauchy mutation, clustering, continuous optimization

## Abstract

Candidate generation in population-based optimization depends on both the starting population and the information exchanged during search. This study proposes the dual-strategy enhanced cuckoo catfish optimizer (DCCO), which modifies these two stages within the cuckoo catfish optimizer (CCO). Clustering-based Cauchy sampling generates approximately half of the initial population around geometric reference centers, while uniform sampling supplies the remainder. Horizontal and normalized-coordinate vertical crossover then recombine individuals and coordinates after each CCO population sweep. On 29 CEC2017 functions at D=30, DCCO achieves an average rank of 1.7586, the lowest among the nine compared methods. Component comparisons and parameter analysis characterize the integrated configuration. In a static wastewater operational model, the mean operating cost index is 22.63% and 24.64% lower than that of the swift flight optimizer (SFO) and CCO, respectively, under dry conditions. The selected decisions remain feasible under dry and rain loads, while all violate the storm constraints, motivating scenario-constrained decisions. The study evaluates the quality–computation trade-off of the integrated search design and its cost–feasibility behavior within the stated algebraic model.

## 1. Introduction

Optimization is widely used to select model parameters and operating conditions in engineering. The aim is to reduce cost or improve performance while meeting practical constraints. These problems can be difficult because changing one variable may affect several aspects of the system, and the best choice often depends on the values of other variables. When each candidate solution must be assessed using a simulation or model, the number of evaluations also limits the search [1,2,3,4].

Gradient-based methods use derivative information to guide the search toward better solutions. For problems evaluated through simulations or complex models, however, this information may be unavailable or costly to obtain. Derivative-free methods address this difficulty by using objective-function values without requiring derivatives [5]. Population-based methods are one option: they maintain a set of candidate solutions and use the evaluated results to generate and select new candidates. This process allows the search to examine different regions and improve the retained solutions over successive iterations [6,7].

Population-based metaheuristics use different rules to generate and select candidate solutions [8,9,10,11,12]. Storn and Price proposed differential evolution (DE), which uses differences between population members to generate trial solutions [13]. In particle swarm optimization (PSO), proposed by Kennedy and Eberhart, particles update their positions using their own experience and information from other particles [14]. Mirjalili et al. developed the grey wolf optimizer (GWO) by modeling leadership and hunting behaviors [15]. More recently, Zheng et al. proposed moss growth optimization (MGO). It uses wind direction to guide the population and models spore dispersal, propagation, and cryptobiosis to search and retain solutions [16]. These methods use different rules to explore the search space [17].

The no-free-lunch (NFL) theorem shows that no search algorithm has an overall advantage when performance is averaged uniformly over all objective functions in its finite-search setting [18]. For a particular problem, the choice of search rules therefore matters. This motivates the examination of an algorithm’s strengths and weaknesses for the problems under study.

Wang et al. proposed the cuckoo catfish optimizer (CCO) in 2025 [19]. This swarm-intelligence method models the behavior of cuckoo catfish. It uses space compression and surround search to explore the search space, together with predation and replacement to update solutions. During the transition stage, some individuals move toward the best solution while others move away from it. These different moves allow CCO to search broadly and refine solutions locally. The original study reported competitive results on numerical benchmarks and engineering design problems.

CCO still faces several difficulties. Its authors noted that performance depends on parameter settings and that the algorithm can take a relatively long time to run. They also called for further tests on highly constrained problems [19]. Later studies reported difficulties in refining solutions locally and keeping individuals spread across the search space [20,21]. When many individuals gather in a small region, the search may repeatedly produce similar solutions and miss alternatives elsewhere. This raises the question of how to choose starting points and help individuals exchange information as the search proceeds.

Researchers have introduced several changes to improve CCO. Li and Yin proposed DBRCCO, which adds a local search inspired by dung beetle foraging [20]. It also reflects candidates back into the search space when they cross a bound. These changes aim to refine solutions and preserve useful movement near the bounds. Chen et al. proposed ALTCCO, which uses crossover to exchange information between candidates [21]. Its LightTrack strategy stores previous positions and triggers jumps when progress stops. It also adjusts mutation according to how candidates rank in the population. These methods show several ways to change CCO’s search steps and respond when the search stalls.

Two aspects of CCO motivate the changes made in this study. CCO initializes its population by uniform random sampling [19]. With a limited population, some regions may receive few starting points, and the search may miss useful regions early in the run. Later, if individuals become too similar, further updates may produce similar candidates and the search can stall [20,21]. These difficulties motivate an initializer that samples around several reference locations and a search step that recombines individuals and coordinates to generate additional candidates [22].

To address these difficulties, we propose the dual-strategy enhanced cuckoo catfish optimizer (DCCO). It combines clustering-based Cauchy initialization with crisscross search. The two strategies aim to improve initial sampling and help the population continue searching for alternatives during later iterations.

The proposed method is considered in a wastewater operational case study. The aim is to reduce operating cost while keeping effluent concentrations within prescribed limits. Studies using Benchmark Simulation Model No. 1 (BSM1) assess both cost and effluent quality [23,24,25]. Here, a BSM1-inspired algebraic model relates oxygen and nitrate setpoints and sludge flows to these outcomes. Selected operating points are checked under dry, rain, and storm loads to see whether they continue to meet the limits. The case study thus examines cost and feasibility under changing loads within a static model.

DCCO modifies CCO at two stages. Its initializer samples approximately half of the starting population around geometric cluster centers using Cauchy perturbations and retains a uniform component. After each CCO sweep, HCS recombines individuals and VCS recombines normalized coordinates. This arrangement places geometric sampling information at initialization and population/coordinate information in subsequent candidate generation.

DBRCCO [20] adds local search and reflective boundary handling, while ALTCCO [21] combines cross-interaction with historical tracking and adaptive mutation. DCCO instead couples a clustering-centered initializer with a specified post-sweep HCS/VCS sequence. These differences distinguish the source and timing of the additional search information and define the mechanisms compared in the evaluation.

The contributions of this study are summarized as follows:

- DCCO connects clustering-centered Cauchy initialization with post-sweep horizontal and normalized-coordinate vertical crossover. The formulation specifies the sampling, snapshot-based recombination, selection and objective-evaluation accounting.
- The 29-function comparison places DCCO first by average rank within the evaluated method set. Component configurations and joint parameter comparisons characterize the integrated design, alongside its computational cost.
- The wastewater case study relates operating cost to effluent feasibility across dry, rain and storm loads in a static model. A scenario-constrained formulation and an analytical reference clarify the operating margins needed under increased load.

Section 2 describes CCO, and Section 3 presents DCCO and its computational complexity. Section 4 covers the numerical comparison, component analysis, and parameter sensitivity. Section 5 examines wastewater operational optimization, and Section 6 summarizes the study and its limitations.

## 2. Original Cuckoo Catfish Optimizer

CCO was introduced by Wang et al. [19] as a population-based method for continuous optimization. Its biological analogy combines prey encirclement, predation, brood parasitism, and individual replacement. The mathematical procedure contains compressed-space and surround-search operators, a transition operator, chaotic predation, and death-related replacement. CCO selects these operators within each individual update.

### 2.1. Population Representation and Initialization

Consider the bounded minimization problem

(1) $$\min_{x\in \Omega }f\left(x\right),\;\Omega =\prod_{j=1}^{D}\left[l_{j},u_{j}\right],\;l_{j}<u_{j}.$$

Here, *D* is the number of decision variables, *f* is the objective, and $l_{j}$ and $u_{j}$ are the bounds of coordinate *j*. CCO maintains *N* candidate vectors, $x_{i}\in R^{D}$. Its original initialization samples each coordinate uniformly:

(2) $$x_{i,j}=l_{j}+r_{i,j}\left(u_{j}-l_{j}\right),\;r_{i,j}∼U\left(0,1\right).$$

The objective values identify the current best and worst individuals. The algorithm also maintains quantities associated with aggregation, search-operator usage, and unsuccessful updates. Uniform sampling selects initial positions across the feasible box independently of their objective values.

### 2.2. Compressed-Space and Surround Search

Compressed-space search constructs proposals from the current individual, the best position, and sampled population members. One compressed-space update is

(3) $$y_{i}=x_{i}+z\left|g\right|\left(\frac{x_{\mathrm{best}}+x_{r_{1}}}{2}-x_{r_{2}}\right)+\frac{\eta }{2}\left(x_{r_{3}}-x_{r_{4}}\right),$$

where z∈{0,1} is a scalar switch, g∼N(0,1), η∼U(0,1), and $r_{1},\ldots ,r_{4}$ index sampled individuals. Two further compression forms and a self-preservation procedure are specified in the original paper [19] (Eqs. (4)–(5) and Algorithm 1).

Surround search provides spiral and spherical proposals. The spiral mechanism uses relative position and aggregation information, whereas the spherical mechanism selects a reference from the best three individuals and the population mean. The search operators are adjusted according to their usage. The iteration-dependent factors include

(4) $$C^{\left(t\right)}=1-\frac{t}{T_{\max}},\;T_{s}^{\left(t\right)}={\left[1-\sin\left(\frac{\pi t}{2T_{\max}}\right)\right]}^{t/T_{\max}},\;w^{\left(t\right)}=1-\frac{\exp(t/T_{\max})-1}{e-1},$$

where *t* is the current iteration and $T_{\max}$ is the iteration limit. The symbols $T_{s}^{\left(t\right)}$ and $w^{\left(t\right)}$ denote the contraction factor and surrounding-radius factor, respectively.

### 2.3. Transition, Predation, and Replacement

The transition operation uses directions toward or away from the best position and includes a Lévy-related term. Chaotic predation selects a proposal according to the individual’s aggregation relative to an initial population reference. Its three cases combine displacement from the best individual with either no additional chaotic term or Cauchy-shaped and Gaussian coefficients. The Cauchy-shaped coefficient in this baseline operation is distinct from drawing Cauchy random variables for the proposed initialization.

The death and parasitic operations generate replacement proposals either from a region associated with the best solution or from the original search bounds. CCO then applies strict objective-based replacement. Writing $y_{i}$ for the complete candidate produced by the applicable baseline operations,

(5) $$x_{i}^{+}=\left\{\begin{array}{cc} y_{i}, & f\left(y_{i}\right)<f\left(x_{i}\right),\\ x_{i}, & \mathrm{otherwise}. \end{array}\right.$$

CCO resets or increments an unsuccessful-update counter according to this comparison and includes a conditional guidance update to the worst individual. After initialization and evaluation, position updates, replacement, and population-state updates repeat within the search loop. The algorithm returns the best evaluated solution at termination.

DCCO modifies the uniform initialization and introduces a crisscross stage after each population update. The existing CCO search and replacement operators form the basis of its iterative search.

## 3. The Proposed Dual-Strategy Enhanced Cuckoo Catfish Optimizer

DCCO modifies two stages of CCO. Clustering with Cauchy perturbation changes the initial sampling distribution, while crisscross search recombines candidate coordinates after each population update. The two modifications therefore act on the starting population and the subsequent search, respectively.

Recent differential-evolution studies provide useful context for separating population distribution from candidate generation. Covariance-based indicators and targeted perturbations identify and update stagnant individuals [26,27], while hierarchical populations and adaptive cluster division change which individuals contribute to mutation [28,29]. Other approaches combine opposition learning, archive management, or restarting to replenish search diversity [30,31,32]. Multi-stage parameter adaptation and diversity-triggered intervention adjust search behavior over time [33]; dimensional interchange and bidirectional crossover instead modify how coordinate information enters trial solutions [34,35]. These studies motivate distinguishing the role of each operator when interpreting a combined method.

### 3.1. Clustering and Cauchy Mutation Initialization

Clustering-based Cauchy initialization combines cluster centers with heavy-tailed perturbations [36]. A set of candidate locations is partitioned by *K*-means. Cauchy perturbations around the resulting centers generate approximately half of the initial population, and uniform sampling generates the remainder.

Let $S={\left\{s_{i}\right\}}_{i=1}^{M}$ denote the clustering samples. The clustering objective is

(6) $$\min_{\left\{S_{k}\right\},\left\{z_{k}\right\}}\sum_{k=1}^{K}\sum_{s_{i}\in S_{k}}{∥s_{i}-z_{k}∥}_{2}^{2},$$

where *K* is the number of clusters, *M* is the sample size, and $z_{k}$ is the center of cluster $S_{k}$. Equation (6) groups samples by their geometric proximity, independently of their objective values.

Cauchy mutation generates a candidate around a cluster center in coordinate form as

(7) $$R_{i,j}^{\mathrm{new}}=z_{a(i),j}+\xi_{i,j},\;\xi_{i,j}∼\mathrm{Cauchy}\left(\mu ,\sigma \right),\;\sigma >0,$$

where a(i)∈{1,…,K} assigns a cluster center to individual *i*, μ is the location parameter, and σ is the scale. This sampler generates $N_{c}\approx N/2$ individuals. The remaining $N-N_{c}$ individuals follow (2). The proposal is passed through a bound-handling operation $B:R^{D}\to \Omega$ before evaluation.

Cauchy perturbations permit both small displacements and occasional long jumps. Related optimizers use Cauchy mutation to perturb retained solutions or combine it with Gaussian and differential mutation [37,38]. Their scale determines how frequently proposals lie far from a center, while bound handling determines where those proposals finally enter the feasible region. For heterogeneous coordinate ranges, coordinate scaling determines the relative perturbation size in each dimension.

### 3.2. Crisscross Strategy

Crisscross search combines horizontal recombination between individuals with vertical recombination between coordinates [39,40]. In DCCO, horizontal crossover search (HCS) and vertical crossover search (VCS) are applied in sequence after each CCO population update. Greedy competition selects the offspring retained by each operation.

Recent crisscross variants apply information exchange within moss-growth search [41] and within a beaver-behavior optimizer [42], whereas a hybrid snake optimizer studies crisscross learning under structural constraints [43]. These implementations provide related examples of the mechanism; the sampling distribution, activation rule, normalization, and evaluation accounting used here are specified below.

#### 3.2.1. Horizontal Crossover Search

At the beginning of HCS, a random permutation π of {1,…,N} is generated. Consecutive entries form ⌊N/2⌋ disjoint pairs, with i=π(2b−1) and k=π(2b) for pair *b*. If *N* is odd, the final unpaired individual passes through this stage unchanged. A snapshot of the population is used to construct both offspring, so accepting one offspring cannot change the parent used to construct the other. For each coordinate *j*, fresh independent coefficients $r_{1,j},r_{2,j}∼U\left(0,1\right)$ and $c_{1,j},c_{2,j}∼U\left(-1,1\right)$ produce

(8) $$\begin{array}{cc} H_{i,j} & =r_{1,j}x_{i,j}+\left(1-r_{1,j}\right)x_{k,j}+c_{1,j}\left(x_{i,j}-x_{k,j}\right), \end{array}$$

(9) $$\begin{array}{cc} H_{k,j} & =r_{2,j}x_{k,j}+\left(1-r_{2,j}\right)x_{i,j}+c_{2,j}\left(x_{k,j}-x_{i,j}\right). \end{array}$$

The sampling coefficients are also independent across pairs. Equations (8) and (9) can extrapolate beyond the interval between the two parents. Greedy selection then compares each offspring with its parent.

Once all coordinates of an offspring have been generated, its components are clipped to the bounds, $B{\left(y\right)}_{j}=\min\left\{u_{j},\max\left\{l_{j},y_{j}\right\}\right\}$. The complete repaired vector is evaluated once and replaces its own parent only if its objective value is strictly smaller. Ties retain the parent.

#### 3.2.2. Vertical Crossover Search

VCS starts from the population retained after HCS. A dimension permutation ρ and a single random value $p_{v}∼U\left(0,1\right)$ are generated for the whole stage. VCS is active only when $p_{v}<0.6$. The same draw governs all dimension pairs. Consecutive entries of ρ define $j_{1}=\rho \left(2b-1\right)$ and $j_{2}=\rho \left(2b\right)$ for b=1,…,⌊D/2⌋. An unpaired coordinate is retained when *D* is odd, and VCS is omitted when D<2.

To combine variables with different units, the post-HCS population is normalized as ${\tilde{x}}_{i,j}=\left(x_{i,j}-l_{j}\right)/\left(u_{j}-l_{j}\right)$. For each individual and pair, an independent $r_{3,i,b}∼U\left(0,1\right)$ gives

(10) $${\tilde{v}}_{i,j_{1}}=r_{3,i,b}{\tilde{x}}_{i,j_{1}}+\left(1-r_{3,i,b}\right){\tilde{x}}_{i,j_{2}}.$$

The first coordinate $j_{1}$ in each ordered pair receives the new value; the second coordinate and any unpaired coordinate keep their post-HCS values. Starting from ${\tilde{v}}_{i}={\tilde{x}}_{i}$, all active pairs are written into one candidate vector using the unchanged post-HCS snapshot. The candidate in the original variables is then

(11) $$v_{i,j}=l_{j}+\left(u_{j}-l_{j}\right){\tilde{v}}_{i,j},\;j=1,\ldots ,D.$$

When all coordinates share the same bounds, this construction is algebraically equivalent to applying the convex combination in the original coordinates. Normalization also permits the operation to be applied to variables with heterogeneous bounds.

After all dimension pairs have been updated, the candidate is repaired and evaluated once. It replaces the current individual only if its objective value is lower. An inactive stage performs neither candidate evaluations nor replacements.

The stage uses a scalar draw $p_{v}∼U\left(0,1\right)$ and is activated when $p_{v}<q_{v}$, with the fixed empirical setting $q_{v}=0.6$. A single draw governs the complete stage, so all coordinate pairs share the activation event. The parameter therefore controls the expected frequency of VCS calls.

### 3.3. Implementation Framework of DCCO

**Figure 1:**
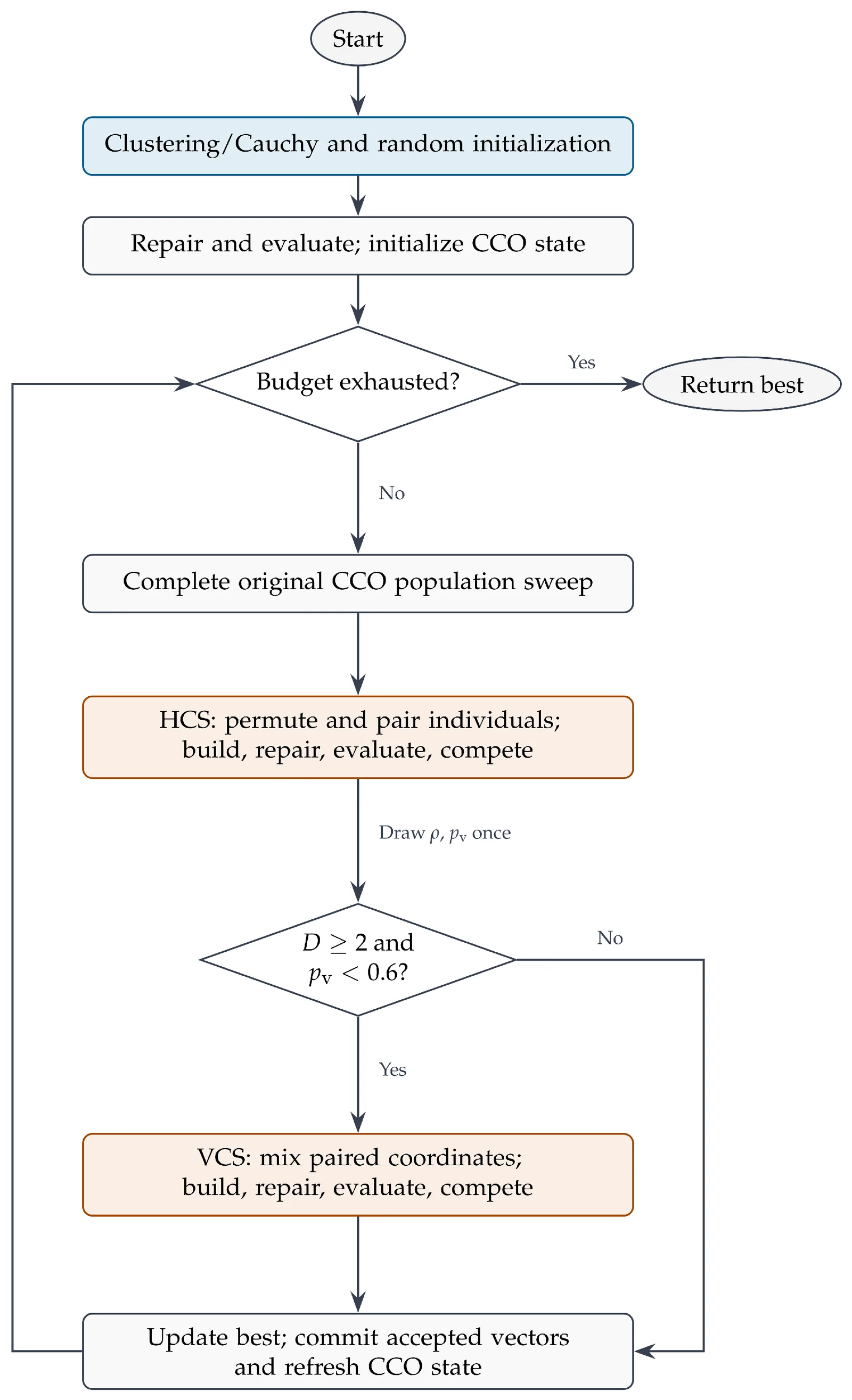
Detailed architectural flowchart of the proposed DCCO algorithm, illustrating the integration of clustering-based Cauchy initialization and crisscross search.

DCCO begins with clustering/Cauchy initialization and evaluates the resulting population. Each iteration then performs the CCO position updates, one horizontal crossover pass, and a vertical crossover pass with probability 0.6. Accepted offspring replace their parents, and the corresponding objective values and best-so-far solution are updated. Population-dependent quantities are refreshed before the next iteration. Algorithm 1 summarizes the procedure, and Figure 1 shows its main stages.
**Algorithm 1** Dual-Strategy Enhanced Cuckoo Catfish Optimizer (DCCO)**Require:** Objective *f*, bounds, *N*, *D*, budget B≥N, initializer and original CCO settings**Ensure:** Best evaluated point $x_{\mathrm{best}}$  1:Set $n_{\mathrm{FE}}\leftarrow 0$; construct the clustering sample and centers  2:Generate $N_{c}\approx N/2$ Cauchy proposals and $N-N_{c}$ uniform proposals  3:**for** each initial candidate **do**  4:      Repair and evaluate it, checking the budget before every objective call  5:      Update $n_{\mathrm{FE}}$ and the best-so-far record  6:**end for**  7:Initialize the retained CCO state and cached objective values  8:**while** $n_{\mathrm{FE}}<B$ **do**  9:      Complete one CCO population sweep with a budget guard before every evaluation10:      **if** the budget is exhausted **then**11:            **return** $x_{\mathrm{best}}$12:      **end if**13:      Execute HCS once on the population14:      **if** HCS reports budget exhaustion **then**15:            **return** $x_{\mathrm{best}}$16:      **end if**17:      Attempt VCS once on the post-HCS population18:      **if** VCS reports budget exhaustion **then**19:            **return** $x_{\mathrm{best}}$20:      **end if**21:      Commit accepted vectors and cached values; refresh the retained CCO state22:**end while**23:**return** $x_{\mathrm{best}}$The budget includes any evaluated clustering samples and all rejected proposals. An exhaustion flag ends the complete execution immediately.

Initialization and all trial evaluations, including those of rejected offspring, count toward the budget. The algorithm terminates when the budget *B* is exhausted and returns the best evaluated solution.

### 3.4. Complexity Analysis

The computational complexity of DCCO depends on the population size *N*, dimension *D*, and number of search iterations T≥1. Clustering *M* sample points into *K* groups over *L* clustering iterations requires O(LMKD) operations, and generating the initial population costs O(ND). During each search iteration, the CCO position updates and the horizontal and vertical crossover operations each require O(ND) operations. The fixed activation probability of vertical crossover affects the constant factor but not the asymptotic order. The total time complexity, excluding objective-function evaluations, is therefore O(LMKD+ND+TND)=O(LMKD+TND). Crisscross search preserves the O(ND) per-iteration cost, while clustering adds a one-time preprocessing cost. When LMK=O(TN), the total complexity reduces to O(TND).

Objective costs must be added explicitly. If $B_{\mathrm{act}}$ is the number of actual objective calls and $C_{f}\left(x_{b}\right)$ the cost of call *b*, the full cost includes $\sum_{b=1}^{B_{\mathrm{act}}}C_{f}\left(x_{b}\right)$. A sorting-based population update additionally contributes O(TNlogN); this term cannot be omitted unless a linear selection procedure is specified. Storage is O(MD+KD+ND) for the clustering sample, centers, population and candidate snapshots, excluding saved traces and objective-specific data. A single inactive VCS stage has zero VCS objective calls; an active stage has at most *N*, subject to the remaining budget. HCS has at most 2⌊N/2⌋ calls. All are charged to $B_{\mathrm{act}}$.

## 4. Experimental Results and Analysis

### 4.1. Benchmark Protocol and Comparative Performance

Benchmark composition and evaluation budgets can change algorithm rankings [44]. Methodological reviews also emphasize testing operators and avoiding claims based only on a biological metaphor [45]. The numerical comparison, component analysis, and parameter study below are interpreted within their stated settings.

The comparison covers the 29 CEC2017 functions listed in Table 1, with F2 excluded. The dimension is D=30, the population size is 30, and each algorithm–function combination has 30 runs with a limit of 300,000 optimization evaluations. The error is the objective value minus the function optimum. Mean errors and sample standard deviations use all 30 runs without a numerical cutoff, and the overall ranking averages the function-wise midranks of those means.

The comparison includes DCCO (Section 3), CCO [19], crested porcupine optimizer (CPO) [46], electrical storm optimization (ESO) [47], swift flight optimizer (SFO) [48], status-based optimization (SBO) [49], DE [13], PSO [14], and GWO [15]. Table 2 reports every function and appends the overall ranking and mean runtime. DCCO has an average rank of 1.7586, followed by CPO at 2.2069 and SBO at 3.1034. Performance varies across functions: CPO has a lower mean on F4 and F27, while DE has a lower mean on F5 and F16. Across the complete function set, the mean-level comparison against CPO is 17/0/12, and against DE it is 27/0/2. These counts denote lower, equal, and higher mean errors from DCCO’s perspective.

The evaluation uses F1 and F3–F30. F2 is omitted consistently with the benchmark organizer’s CEC2017 comparison report (slide 8) [50], which identifies instability, especially at higher dimension, and variation between MATLAB (R2016b) and C implementations (experimental simulations in this study were conducted using MATLAB R2025b, The MathWorks, Natick, MA, USA). The exclusion is common to all methods and is independent of the observed DCCO ranking.

The reported comparison uses D=30, N=30, 30 runs and a total budget of 300,000 objective evaluations per run. Shared initial populations and matched random inputs are unavailable in the submitted comparison, and its run-level tests accordingly treat samples as independent. Each method retains its specified initialization. All evaluated candidates, including rejected proposals and any evaluated preprocessing samples, count toward the same budget. A shared integer seed alone does not imply matching random streams across different algorithms.

### 4.2. Statistical Comparison and Convergence

Table 3 reports adjusted two-sided Mann–Whitney *U* *p*-values. The within-function comparisons treat the samples as independent because shared initial populations and matched random inputs are unavailable. Holm correction is applied jointly to the 232 function–comparator tests at a significance level of 0.05. For example, the adjusted *p*-value against CPO on F30 is 0.0128, whereas the corresponding value on F19 is 1.0. A small adjusted *p*-value provides evidence of a distributional difference; the mean errors indicate the direction of the comparison.

**Figure 2:**
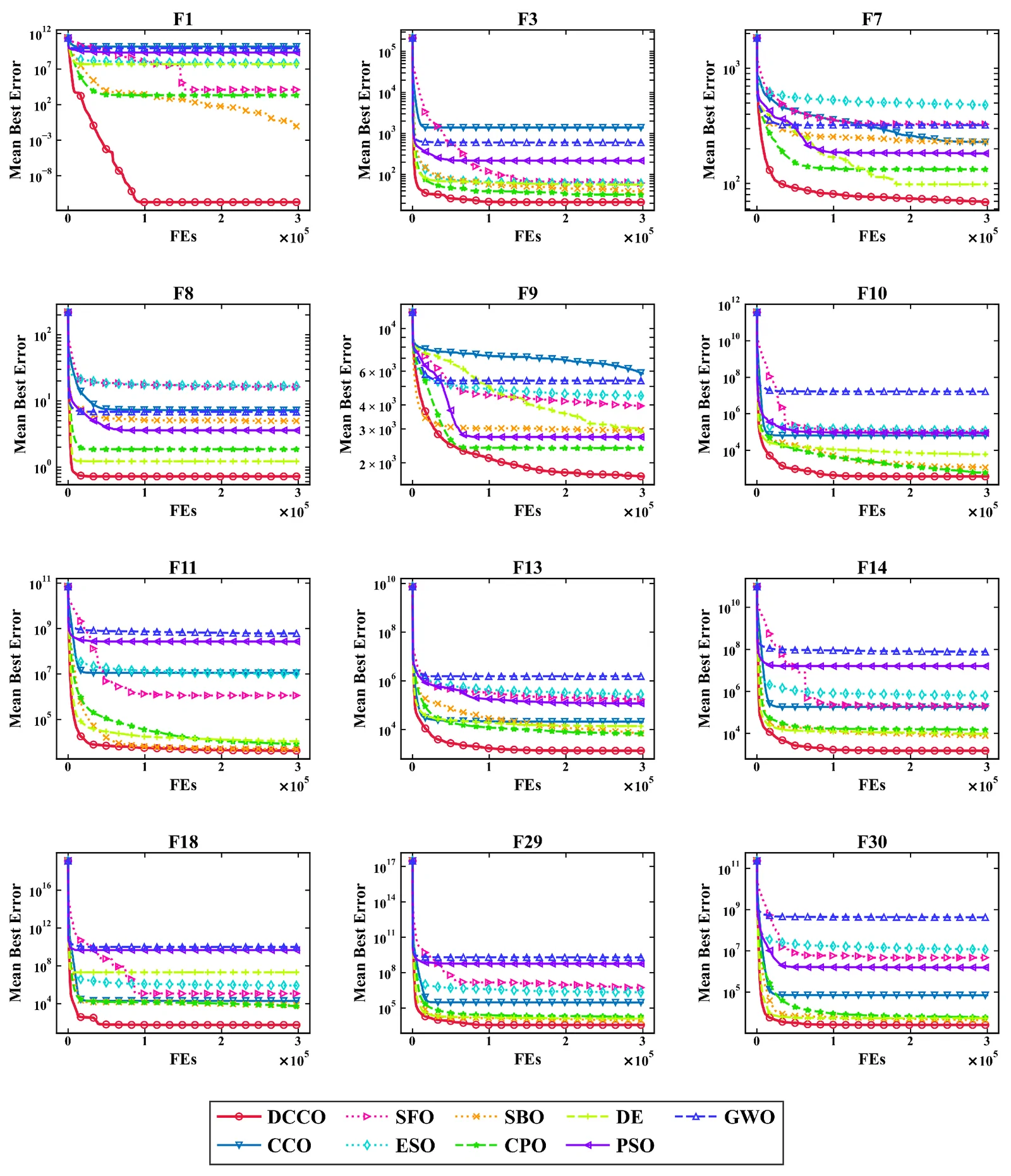
Convergence behavior analysis on twelve representative benchmark functions.

Figure 2 displays twelve functions with visible separation among the curves. The horizontal axis is cumulative objective evaluations. The vertical axis shows the mean best error across all 30 runs on a logarithmic scale; the display floor is applied before averaging and does not enter the final-error statistics. The curves show a rapid error reduction on F1 and separation on F3 and F7, while they are more closely grouped on F11 and F29. The complete 29-function table remains the basis for the aggregate comparison.

### 4.3. Component Ablation and Joint Parameter Sensitivity

The four configurations in Table 4 compare the two modifications separately and in combination. CCO-I changes the initializer, CCO-CC adds the HCS–VCS search stage, and DCCO includes both. DCCO attains the lowest mean on 18 of the 29 cases and has an average rank of 1.5862, compared with 2.9310 for CCO-I, 2.6897 for CCO-CC, and 2.7931 for CCO. After multiple-testing correction, the win/tie/loss counts relative to DCCO are 3/5/21 for CCO-I, 1/8/20 for CCO-CC, and 6/2/21 for CCO. The combined DCCO configuration achieves the best overall ranking across the benchmark functions, supporting the combined configuration within this comparison, without identifying a causal interaction between its operators.

The joint sensitivity design varies the cluster count *K*, Cauchy scale σ, and clustering sample count *M*. All 60 configurations are ranked on each of 29 cases after averaging 30 repetitions; Response is the mean of these ranks. Table 5 reports the ten configurations with the smallest Responses. Design 53, with K=5, σ=0.1466, and M=80, has a Response of 5.3103. Design 26 follows with a Response of 10.2414. Since the parameters vary jointly, this ordering does not identify the independent effect of any single parameter and does not establish a universally optimal setting.

### 4.4. Across-Function Inference and Effect-Size Interpretation

An across-function analysis of the 29-by-9 mean-error table gives a Friedman statistic of 171.6782 with eight degrees of freedom ($p=5.7376\times 10^{-33}$). Two-sided normal-approximation comparisons of average ranks against DCCO, adjusted jointly by Holm across eight contrasts, distinguish DCCO from six comparators; differences from CPO ($p_{\mathrm{adj}}=0.5331$) and SBO ($p_{\mathrm{adj}}=0.1230$) are not significant. Functions are the blocks in this analysis, while the existing independent-sample tests assess within-function differences. This analysis follows the rank-comparison framework in [51], and the corresponding results are summarized in Table 6.

The combined configuration has the best aggregate result in the component comparison. Convergence, diversity and robustness characterize different aspects of the search behavior. A dimension-normalized diversity measure is

$$D\left(t\right)={\left[\frac{1}{ND}\sum_{i=1}^{N}\sum_{j=1}^{D}{\left(\frac{x_{i,j}^{\left(t\right)}-{\bar{x}}_{j}^{\left(t\right)}}{u_{j}-l_{j}}\right)}^{2}\right]}^{1/2}.$$

Convergence is compared against consumed objective evaluations. For target-hitting analyses, unsuccessful runs should be retained as censored observations. Configuration rankings describe aggregate performance; attributing a statistical interaction to the components requires a factorial contrast on a pre-specified error scale.

For two independent error samples, a directionally explicit effect size is $A_{\mathrm{lower}}=P\left(E_{\mathrm{DCCO}}<E_{A}\right)+\frac{1}{2}P\left(E_{\mathrm{DCCO}}=E_{A}\right)$. Values greater than 0.5 favor DCCO. A run-resampling confidence interval describes estimation uncertainty; pointwise intervals do not provide simultaneous coverage for every function–comparator pair. The existing Holm-adjusted tests and effect sizes answer related but different questions.

### 4.5. Scalability, Related Variants and Uncertainty

Table 7 compares D=50 and D=100 for DCCO, CCO, DBRCCO [20], and ALTCCO [21]. The target common protocol uses N=30, 30 repetitions and B=10,000D per function, including every evaluated preprocessing or rejected candidate. Appendix B reports every function. The four-method mean ranks for DCCO are 2.0000 and 2.1034. Against CCO, the lower/equal/higher mean counts are 26/0/3 and 20/0/9, respectively; increasing dimension does not preserve a gain on all functions. These ranks belong to this four-method family only.

Distance concentration can affect geometric clustering at higher dimensions. Cauchy proposals and boundary clipping also shape the starting distribution, making initialization diagnostics relevant alongside the function-level comparison.

Table 8 gives independent-sample effects and pointwise 95% intervals for F3 using the same added samples as the full tables. The added multiple-testing family is 174 dimension–function–comparator tests, separate from the original 232 tests. Against ALTCCO at D=100, the point estimate is 0.732 and the interval is [0.587, 0.852], but the adjusted *p* is 0.138. A pointwise interval does not control family-wise errors across all reported contrasts. The data package contains all 174 rows.

### 4.6. Runtime, Component Diagnostics and Parameter Response

The original mean runtimes are 99.81 s for DCCO and 75.71 s for CCO, a 31.83% increase. Together with the solution-quality comparison, these measurements quantify the computational trade-off of the integrated configuration. Clustering contributes a one-time O(LMKD) cost, and the post-sweep operators add candidate generation, repair and selection. Objective calls and sorting are accounted for separately in the full-cost model. The total timings characterize the complete methods rather than isolated component costs.

The original parameter table reports the ten best configurations from an irregular Latin-hypercube design. The additional sampled-point projections and complete table describe the response across the displayed design. Interpolated slices visualize the joint pattern between these points.

Table 9 compares the global VCS activation probability while keeping other settings fixed in the comparison specification. The displayed rank is smallest at 0.6 and increases at both endpoints. The setting $q_{v}=0$ removes VCS while retaining HCS, isolating the contribution of the vertical stage in this comparison.

**Figure 3:**
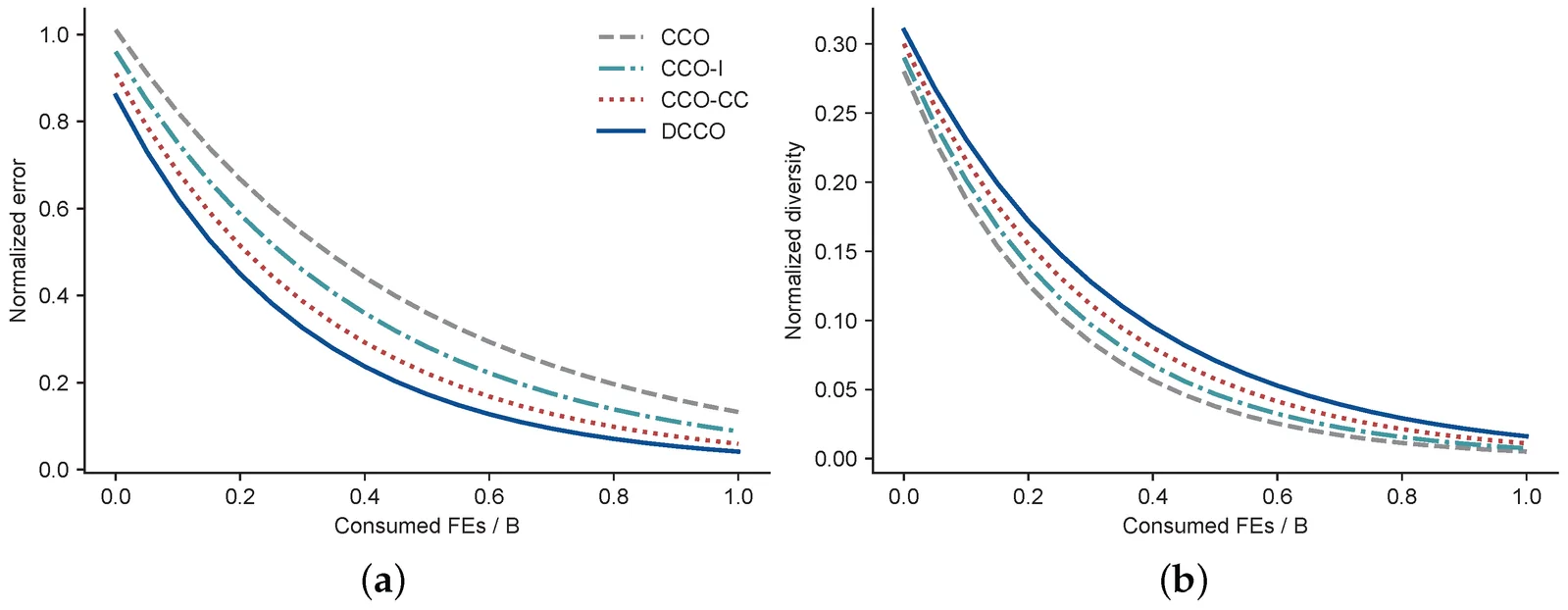
Component convergence and diversity: (**a**) normalized error convergence curves; (**b**) normalized population diversity trajectories. The horizontal axis is a specified fraction of the FE budget; the vertical quantities are normalized diagnostic values.

Figure 3 places component convergence and normalized diversity on the same consumed-budget axis. The four configurations are CCO, initialization-only CCO-I, crossover-only CCO-CC, and DCCO. A lower terminal error can coexist with a smaller diversity measure, so sustained spread alone is not a success criterion. Robustness should also be quantified through run-to-run dispersion and failure probability at fixed target thresholds, with failures retained.

**Figure 4:**
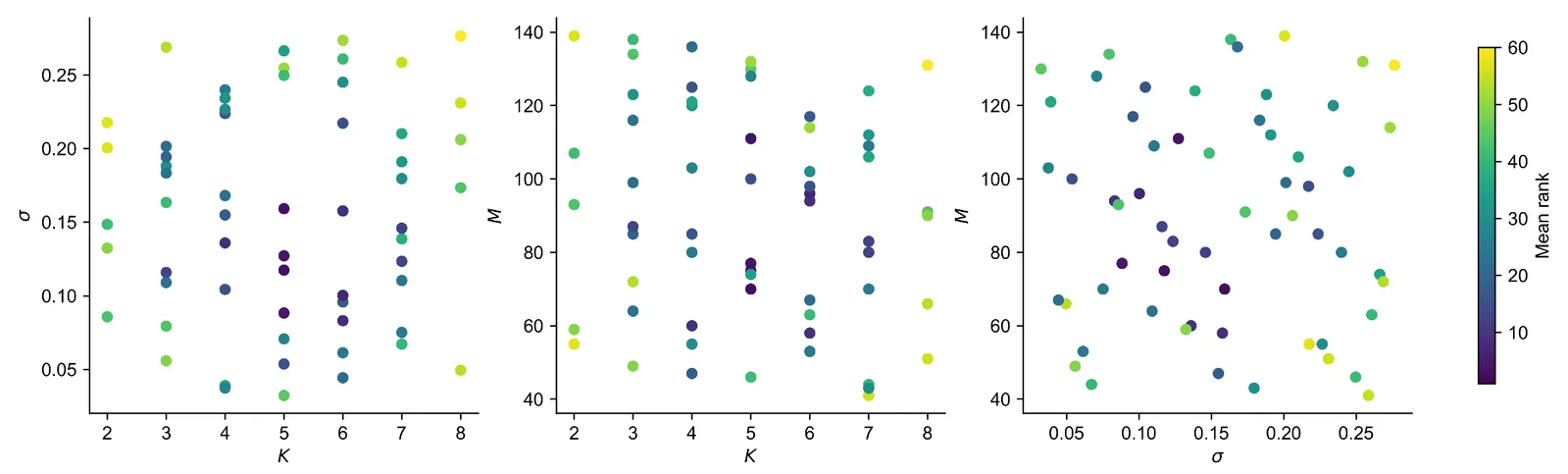
All 60 additional configurations projected onto the three parameter planes. Colors encode the mean within-design rank; lower is better.

**Figure 5:**
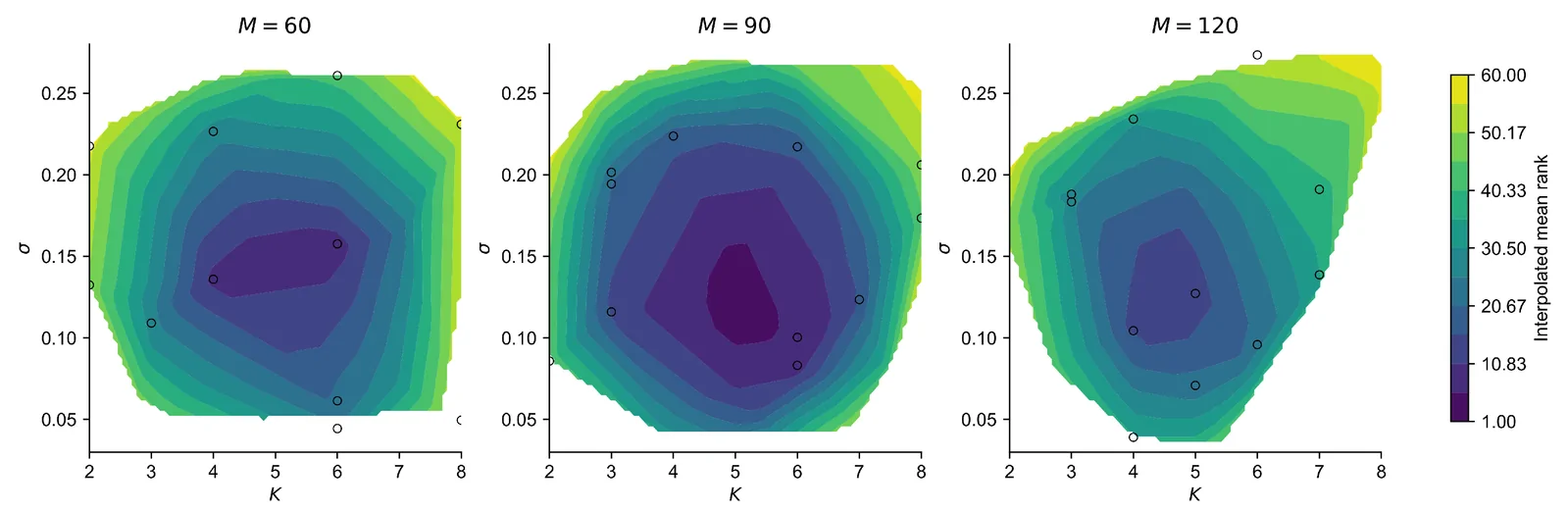
Three interpolated response slices. Colors interpolate ranks linearly; blank regions lie outside the sampled convex hull. Open circles mark sampled points within 10 units of each displayed M slice, so they are nearby projections rather than exact-slice observations.

The three projections in Figure 4 show all 60 points of the additional parameter display. Figure 5 interpolates these points at M=60,90,120 and masks locations outside their convex hull. Since *K* and *M* are discrete settings, interpolation is a visualization aid, not evidence that an untested fractional cluster count is implementable. The response is the mean rank among the 60 displayed configurations over 29 functions, not the nine-algorithm rank in the main comparison. Appendix C contains the complete displayed design.

## 5. Wastewater Operational Optimization

Recent wastewater studies jointly model effluent quality and energy demand using ensemble predictors and multiobjective optimization [52,53]. Bayesian tuning has also been used to improve predictions of total nitrogen and energy consumption [54]. Studies of operating conditions and aeration control show why oxygen settings and recirculation must be assessed against both treatment quality and resource use [55,56].

Dynamic operation adds a separate control problem. Reinforcement-learning reviews discuss implementation and model-integration challenges [57], while recent studies combine reinforcement learning with Bayesian optimization, optimize setpoints with disturbance-rejection control, or update predictive aeration models as operating data change [58,59,60]. A cyclic residual-steered Bayesian framework additionally adjusts oxygen setpoints and chemical dosing under changing influent conditions [61]. The present case study employs a static algebraic operational model and fixed-decision load checks to evaluate operating costs and constraint feasibility under varying influent conditions.

Wastewater operation is represented by the decision vector x=(d,n,r,w), where *d* and *n* are dissolved-oxygen and nitrate setpoints, and *r* and *w* are return-sludge and waste-sludge flows. The respective ranges are [1,3], [0.5,2], [5000,15000], and [50,200]; concentrations are expressed in mg/L and flows in $m^{3}/d$. Coordinate normalization maps these heterogeneous ranges to a unit box before interpreting a perturbation scale. The operating cost index (OCI) is defined by

(12) $$\mathrm{OCI}\left(x\right)=0.8d^{2}+0.6n+0.00012r+0.0204w.$$

For a load multiplier *ℓ*, the corresponding effluent responses are

(13) $$\begin{array}{cc} C_{\mathrm{NH}}\left(x,l\right) & =l\left(1+\frac{5}{d+0.4}+0.08n\right), \end{array}$$

(14) $$\begin{array}{cc} C_{\mathrm{NO}}\left(x,l\right) & =l\left(3+2n+\frac{3000}{r}\right), \end{array}$$

(15) $$\begin{array}{cc} C_{\mathrm{COD}}\left(x,l\right) & =l\left(20+\frac{12}{d+0.3}+\frac{100}{w}\right). \end{array}$$

The imposed upper limits are 4.5, 12, and 60, respectively. The accompanying effluent quality index (EQI) is $\mathrm{EQI}=2C_{\mathrm{NH}}+C_{\mathrm{NO}}+0.1C_{\mathrm{COD}}$. The cost and effluent responses are evaluated at the same decision vector.

Table 10 compares DCCO with CCO [19], CPO [46], ESO [47], SFO [48], SBO [49], DE [13], PSO [14], and GWO [15] under the dry-load setting across 30 independent runs. DCCO has the lowest mean OCI of 3.4849, compared with 4.5043 for SFO and 4.6245 for CCO, giving mean reductions of 22.63% and 24.64%, respectively. Its minimum and maximum OCI are also the lowest in the table. These statistics summarize each method’s outcome distribution.

DCCO has a standard deviation of 0.0255, which is 94.51% lower than that of SFO. Its coefficient of variation is 0.73%, and its OCI ranges from 3.4277 to 3.5463. Even the upper end of this range remains below the mean OCI of every comparator. Together, these statistics show a narrow range of low OCI values across the 30 records.

The selected decision vectors are held fixed under load multipliers l=1, 1.15, and 1.35, corresponding to dry, rain, and storm settings. All 270 selected decisions satisfy the dry-load constraints. Under the rain setting, all 30 DCCO decisions remain feasible, compared with 14 for CPO, 11 for SFO, and 7 for CCO. Under these operating conditions, DCCO combines the lowest dry-load cost with complete feasibility across all its runs under the moderate load increase. At the storm load, none of the DCCO decisions remain feasible, and only three records across the other methods satisfy all constraints. The advantage is limited to the dry and rain settings. The stronger disturbance requires additional operating margins or control adaptation. Since OCI is independent of *ℓ*, these load checks assess effluent feasibility rather than a change in operating cost.

### 5.1. Interpretation of Cost and Variability

Here, operating cost index (OCI) and effluent quality index (EQI) denote the operational performance metrics defined by the case-study formulation. The formulation is benchmarked against key principles of Benchmark Simulation Model No. 1 (BSM1), capturing core operational trade-offs among aeration intensity, pumping energy, and effluent quality constraints within an efficient algebraic surrogate. Given the substantial computational runtime required by iterative population-based metaheuristics across multiple independent runs, this setup provides a computationally tractable platform for rigorous statistical comparison. The percentage reduction relative to method *A* is

$$\Delta_{A}=100\frac{{\bar{\mathrm{OCI}}}_{A}-{\bar{\mathrm{OCI}}}_{\mathrm{DCCO}}}{{\bar{\mathrm{OCI}}}_{A}}.$$

Using the 30-run means yields reductions of 22.6317% against SFO and 24.6427% against CCO, demonstrating clear operational efficiency gains under standard dry-load conditions. The accompanying fixed-decision rain and storm checks further evaluate effluent feasibility and operating robustness under influent shock loads.

The small OCI standard deviation corresponds to a coefficient of variation of approximately 0.7319%, reflecting the high search consistency and solution stability of DCCO. Table 11 organizes a component-wise cost and feasibility comparison. All 30 decisions for each component satisfy the dry constraint, while rain and storm feasibility differentiate the operating margins. While the present algebraic evaluation provides high computational throughput, extending DCCO to full dynamic BSM1 simulation environments with time-varying biological states and feedback control remains an important and promising direction for future work.

### 5.2. Scenario Constraints and an Analytical Reference

A scenario-constrained extension enforces the effluent limits during optimization:

$$\min_{x\in \Omega }\mathrm{OCI}\left(x\right),\;C_{j}\left(x,l\right)\leq L_{j}\;\forall j\in \left\{\mathrm{NH},\mathrm{NO},\mathrm{COD}\right\},\;\forall l\in \left\{1,1.15,1.35\right\}.$$

For a scenario-constrained extension of DCCO, each candidate can be scored by its OCI and a normalized constraint violation,

$$V\left(x\right)=\max_{l\in \{1,1.15,1.35\}}\sum_{j\in \{\mathrm{NH},\mathrm{NO},\mathrm{COD}\}}\max\;\left(0,\frac{C_{j}\left(x,l\right)-L_{j}}{L_{j}}\right).$$

After box repair, selection would prefer a feasible candidate to an infeasible one, lower OCI between two feasible candidates, and lower *V* between two infeasible candidates; equal violations would be resolved by OCI. The same comparator would apply to the CCO, HCS and VCS stages. One candidate assessment would include OCI and all three scenario responses, with scenario calls recorded separately from candidate assessments. This extension changes constraint handling while retaining the candidate-generation operators.

All three effluent responses increase with *ℓ*, so the storm constraint dominates in this static model. Cost is increasing in each decision over the stated bounds. Choosing n=0.5, r=5000 and w=50 minimizes their individual cost terms, and the ammonia constraint imposes

$$d^{*}\left(l\right)=\max\left\{1,\frac{5}{4.5/l-1.04}-0.4\right\}.$$

For the three stated loads, the resulting nitrate and COD values satisfy their limits. Any feasible point must have n≥0.5 and hence $d\geq d^{*}\left(l\right)$, while r≥5000 and w≥50. The constructed point therefore attains the global lower bound on the separable, increasing cost for these loads. At l=1.35 it gives $d^{*}=1.78023256$, OCI =4.45538237, ammonia =4.5, nitrate =6.21 and COD =37.48759.

The construction provides an exact reference for the stated algebraic model, as reported in Table 12. The dry-load decisions lack the operating margin needed at storm load, while the scenario-constrained formulation has a feasible solution for all three loads. Algorithmic evaluation on this formulation should report cost and feasibility jointly; dynamic treatment behavior remains a separate validation setting.

The dry-load analytical minimum is 2.7938, compared with the reported DCCO mean of 3.4849. This exact baseline quantifies the objective-value gap within the static equations. The case study consequently serves two purposes: comparing the operating points produced by the search methods and examining the additional feasibility requirements introduced by increased load.

## 6. Conclusions

DCCO couples clustering-centered Cauchy initialization with post-sweep crisscross search in CCO. The two stages modify the starting distribution and the subsequent use of individual and coordinate information. Across 29 CEC2017 functions at D=30, DCCO achieves an average rank of 1.7586, and the component comparison favors the integrated configuration. The mean runtime of 99.81 s, compared with 75.71 s for CCO, characterizes the additional computational cost associated with this solution-quality result.

In the wastewater benchmark, DCCO reduces mean OCI by 22.63% and 24.64% relative to SFO and CCO under dry load, while maintaining complete feasibility under dry and rain conditions. The scenario-constrained formulation and analytical reference further identify feasible operating margins across all load levels. Building upon the proven search efficiency and decision stability of the proposed approach, future work will integrate DCCO into high-fidelity dynamic BSM1 simulation environments to examine time-varying biological kinetics, aeration controller adaptation, and real-time disturbance rejection.

## Figures and Tables

**Table 1:** CEC2017 benchmark functions.

Function	Function Name	Class	Optimum
F1	Shifted and Rotated Bent Cigar Function	Unimodal	100
F3	Shifted and Rotated Zakharov Function	Unimodal	300
F4	Shifted and Rotated Rosenbrock’s Function	Multimodal	400
F5	Shifted and Rotated Rastrigin’s Function	Multimodal	500
F6	Shifted and Rotated Expanded Scaffer’s F6 Function	Multimodal	600
F7	Shifted and Rotated Lunacek Bi-Rastrigin Function	Multimodal	700
F8	Shifted and Rotated Non-Continuous Rastrigin’s Function	Multimodal	800
F9	Shifted and Rotated Lévy Function	Multimodal	900
F10	Shifted and Rotated Schwefel’s Function	Multimodal	1000
F11	Hybrid Function 1 (N = 3)	Hybrid	1100
F12	Hybrid Function 2 (N = 3)	Hybrid	1200
F13	Hybrid Function 3 (N = 3)	Hybrid	1300
F14	Hybrid Function 4 (N = 4)	Hybrid	1400
F15	Hybrid Function 5 (N = 4)	Hybrid	1500
F16	Hybrid Function 6 (N = 4)	Hybrid	1600
F17	Hybrid Function 7 (N = 5)	Hybrid	1700
F18	Hybrid Function 8 (N = 5)	Hybrid	1800
F19	Hybrid Function 9 (N = 5)	Hybrid	1900
F20	Hybrid Function 10 (N = 6)	Hybrid	2000
F21	Composition Function 1 (N = 3)	Composition	2100
F22	Composition Function 2 (N = 3)	Composition	2200
F23	Composition Function 3 (N = 4)	Composition	2300
F24	Composition Function 4 (N = 4)	Composition	2400
F25	Composition Function 5 (N = 5)	Composition	2500
F26	Composition Function 6 (N = 5)	Composition	2600
F27	Composition Function 7 (N = 6)	Composition	2700
F28	Composition Function 8 (N = 6)	Composition	2800
F29	Composition Function 9 (N = 3)	Composition	2900
F30	Composition Function 10 (N = 3)	Composition	3000

**Table 2:** Results of the DCCO and Other Algorithms on CEC2017 Benchmark Functions. Bold values indicate the best performance for each benchmark function.

	F1		F3		F4	
Algo.	Avg	Std	Avg	Std	Avg	Std
DCCO	$1.0923\times 10^{-12}$	$5.2589\times 10^{-12}$	$2.1209\times 10^{1}$	$1.5250\times 10^{1}$	$5.6208\times 10^{1}$	$1.9830\times 10^{1}$
DE	$4.8522\times 10^{7}$	$1.3522\times 10^{8}$	$5.6479\times 10^{1}$	$2.9283\times 10^{1}$	$1.3929\times 10^{2}$	$2.5693\times 10^{2}$
CCO	$1.4148\times 10^{10}$	$5.8367\times 10^{9}$	$1.3898\times 10^{3}$	$7.8120\times 10^{2}$	$2.0813\times 10^{4}$	$8.0590\times 10^{3}$
GWO	$8.5275\times 10^{9}$	$8.3075\times 10^{8}$	$6.0229\times 10^{2}$	$9.4657\times 10^{1}$	$7.2525\times 10^{3}$	$1.1465\times 10^{3}$
PSO	$2.1680\times 10^{9}$	$2.1113\times 10^{9}$	$2.1712\times 10^{2}$	$2.1490\times 10^{2}$	$4.8894\times 10^{3}$	$3.0901\times 10^{3}$
CPO	$2.0885\times 10^{3}$	$2.5943\times 10^{3}$	$3.1087\times 10^{1}$	$1.7409\times 10^{1}$	$4.7583\times 10^{1}$	$1.6138\times 10^{1}$
ESO	$6.3924\times 10^{7}$	$1.0112\times 10^{7}$	$6.0635\times 10^{1}$	$2.8756\times 10^{1}$	$3.7025\times 10^{2}$	$3.0258\times 10^{1}$
SFO	$1.2728\times 10^{4}$	$7.6938\times 10^{3}$	$6.1557\times 10^{1}$	$5.5187\times 10^{1}$	$5.1649\times 10^{2}$	$1.1468\times 10^{2}$
SBO	$7.0572\times 10^{-2}$	$2.0430\times 10^{-1}$	$3.8721\times 10^{1}$	$2.7851\times 10^{1}$	$2.2695\times 10^{2}$	$6.3126\times 10^{1}$
	**F5**		**F6**		**F7**	
**Algo.**	**Avg**	**Std**	**Avg**	**Std**	**Avg**	**Std**
DCCO	$1.7840\times 10^{-5}$	$8.5526\times 10^{-5}$	$9.7505\times 10^{1}$	$2.0249\times 10^{1}$	$6.8878\times 10^{1}$	$1.2245\times 10^{1}$
DE	$1.0631\times 10^{-6}$	$2.1742\times 10^{-6}$	$1.0965\times 10^{2}$	$6.6298\times 10^{1}$	$9.8440\times 10^{1}$	$2.5279\times 10^{1}$
CCO	$1.7750\times 10^{-3}$	$1.1789\times 10^{-3}$	$8.1837\times 10^{5}$	$2.8249\times 10^{5}$	$2.2843\times 10^{2}$	$3.8849\times 10^{1}$
GWO	$5.7283\times 10^{-3}$	$1.7307\times 10^{-3}$	$2.8739\times 10^{5}$	$3.8307\times 10^{4}$	$3.2116\times 10^{2}$	$3.0909\times 10^{1}$
PSO	$5.8987\times 10^{-5}$	$8.3707\times 10^{-5}$	$1.5801\times 10^{5}$	$1.0196\times 10^{5}$	$1.8233\times 10^{2}$	$4.8665\times 10^{1}$
CPO	$3.6835\times 10^{-5}$	$8.5493\times 10^{-5}$	$1.3953\times 10^{2}$	$1.3909\times 10^{1}$	$1.3275\times 10^{2}$	$2.6210\times 10^{1}$
ESO	$2.4137\times 10^{-3}$	$2.7279\times 10^{-3}$	$2.7058\times 10^{3}$	$4.0610\times 10^{2}$	$4.8021\times 10^{2}$	$8.8835\times 10^{1}$
SFO	$9.4987\times 10^{-4}$	$1.2114\times 10^{-3}$	$9.6763\times 10^{2}$	$4.2466\times 10^{2}$	$3.2666\times 10^{2}$	$9.1432\times 10^{1}$
SBO	$3.4284\times 10^{-4}$	$5.0838\times 10^{-4}$	$3.0733\times 10^{2}$	$7.0145\times 10^{1}$	$2.3067\times 10^{2}$	$4.8510\times 10^{1}$
	**F8**		**F9**		**F10**	
**Algo.**	**Avg**	**Std**	**Avg**	**Std**	**Avg**	**Std**
DCCO	$7.2067\times 10^{-1}$	$9.5371\times 10^{-1}$	$1.6985\times 10^{3}$	$3.7676\times 10^{2}$	$3.6634\times 10^{2}$	$4.3705\times 10^{2}$
DE	$1.2212\times 10^{0}$	$9.0668\times 10^{-1}$	$2.9020\times 10^{3}$	$9.1428\times 10^{2}$	$6.0009\times 10^{3}$	$5.6742\times 10^{3}$
CCO	$7.2243\times 10^{0}$	$2.2480\times 10^{0}$	$5.8386\times 10^{3}$	$9.3749\times 10^{2}$	$6.4071\times 10^{4}$	$2.4126\times 10^{4}$
GWO	$6.8083\times 10^{0}$	$9.2683\times 10^{-1}$	$5.3476\times 10^{3}$	$4.7622\times 10^{2}$	$1.6434\times 10^{7}$	$6.0369\times 10^{6}$
PSO	$3.5819\times 10^{0}$	$2.5690\times 10^{0}$	$2.7225\times 10^{3}$	$7.0804\times 10^{2}$	$8.9008\times 10^{4}$	$6.1345\times 10^{4}$
CPO	$1.8519\times 10^{0}$	$1.1460\times 10^{0}$	$2.3850\times 10^{3}$	$5.9147\times 10^{2}$	$5.7135\times 10^{2}$	$7.7798\times 10^{2}$
ESO	$1.6770\times 10^{1}$	$6.5600\times 10^{0}$	$4.4653\times 10^{3}$	$6.2305\times 10^{2}$	$1.1422\times 10^{5}$	$4.3700\times 10^{4}$
SFO	$1.6243\times 10^{1}$	$5.7335\times 10^{0}$	$3.9509\times 10^{3}$	$8.1825\times 10^{2}$	$1.0129\times 10^{5}$	$6.4692\times 10^{4}$
SBO	$4.9769\times 10^{0}$	$2.1108\times 10^{0}$	$2.9613\times 10^{3}$	$5.7961\times 10^{2}$	$1.1368\times 10^{3}$	$6.3778\times 10^{2}$
	**F11**		**F12**		**F13**	
**Algo.**	**Avg**	**Std**	**Avg**	**Std**	**Avg**	**Std**
DCCO	$4.2329\times 10^{3}$	$4.0520\times 10^{3}$	$2.9443\times 10^{3}$	$2.9211\times 10^{3}$	$1.3569\times 10^{3}$	$2.9326\times 10^{3}$
DE	$1.1134\times 10^{4}$	$1.2240\times 10^{4}$	$6.8860\times 10^{3}$	$4.6867\times 10^{3}$	$1.3687\times 10^{4}$	$1.5128\times 10^{4}$
CCO	$1.1152\times 10^{7}$	$1.2622\times 10^{7}$	$3.5084\times 10^{6}$	$7.0997\times 10^{6}$	$2.1009\times 10^{4}$	$1.5086\times 10^{4}$
GWO	$6.1156\times 10^{8}$	$2.4633\times 10^{8}$	$4.4238\times 10^{8}$	$1.4709\times 10^{8}$	$1.5435\times 10^{6}$	$1.0731\times 10^{6}$
PSO	$2.6998\times 10^{8}$	$4.4049\times 10^{8}$	$3.3334\times 10^{7}$	$1.0014\times 10^{8}$	$1.1893\times 10^{5}$	$4.4315\times 10^{5}$
CPO	$8.0042\times 10^{3}$	$5.0397\times 10^{3}$	$3.3997\times 10^{3}$	$2.5261\times 10^{3}$	$6.8578\times 10^{3}$	$1.1242\times 10^{4}$
ESO	$1.0284\times 10^{7}$	$3.7481\times 10^{6}$	$2.0771\times 10^{6}$	$6.0460\times 10^{5}$	$2.8052\times 10^{5}$	$1.2394\times 10^{5}$
SFO	$1.1395\times 10^{6}$	$2.4612\times 10^{6}$	$8.5871\times 10^{4}$	$7.4028\times 10^{4}$	$1.7411\times 10^{5}$	$7.8471\times 10^{4}$
SBO	$4.9008\times 10^{3}$	$4.4233\times 10^{3}$	$5.0469\times 10^{3}$	$4.6838\times 10^{3}$	$6.8116\times 10^{3}$	$7.3656\times 10^{3}$
	**F14**		**F15**		**F16**	
**Algo.**	**Avg**	**Std**	**Avg**	**Std**	**Avg**	**Std**
DCCO	$1.5292\times 10^{3}$	$2.1045\times 10^{3}$	$3.8485\times 10^{2}$	$2.6377\times 10^{2}$	$3.5371\times 10^{2}$	$3.5161\times 10^{2}$
DE	$9.5638\times 10^{3}$	$5.6600\times 10^{3}$	$6.9591\times 10^{3}$	$2.6570\times 10^{4}$	$1.8789\times 10^{2}$	$2.0583\times 10^{2}$
CCO	$1.8309\times 10^{5}$	$3.9990\times 10^{5}$	$3.0417\times 10^{3}$	$2.4727\times 10^{3}$	$6.2592\times 10^{3}$	$1.1337\times 10^{4}$
GWO	$7.6074\times 10^{7}$	$4.6163\times 10^{7}$	$5.8937\times 10^{6}$	$2.7219\times 10^{6}$	$4.1267\times 10^{6}$	$1.3707\times 10^{7}$
PSO	$1.5864\times 10^{7}$	$4.4031\times 10^{7}$	$1.6608\times 10^{6}$	$5.4657\times 10^{6}$	$4.6183\times 10^{7}$	$1.9706\times 10^{8}$
CPO	$1.4885\times 10^{4}$	$6.7514\times 10^{3}$	$1.4766\times 10^{2}$	$2.0624\times 10^{2}$	$2.2471\times 10^{2}$	$3.1422\times 10^{2}$
ESO	$6.3707\times 10^{5}$	$2.6095\times 10^{5}$	$1.3692\times 10^{4}$	$1.0286\times 10^{4}$	$1.4728\times 10^{3}$	$4.3790\times 10^{2}$
SFO	$2.0631\times 10^{5}$	$3.3189\times 10^{5}$	$2.2718\times 10^{4}$	$2.1469\times 10^{4}$	$7.1542\times 10^{3}$	$1.2457\times 10^{4}$
SBO	$7.9854\times 10^{3}$	$6.2856\times 10^{3}$	$2.4337\times 10^{2}$	$3.2555\times 10^{2}$	$3.5054\times 10^{2}$	$3.5781\times 10^{2}$
	**F17**		**F18**		**F19**	
**Algo.**	**Avg**	**Std**	**Avg**	**Std**	**Avg**	**Std**
DCCO	$3.3148\times 10^{3}$	$6.1552\times 10^{3}$	$5.5721\times 10^{1}$	$1.0386\times 10^{2}$	$1.7909\times 10^{2}$	$1.6618\times 10^{2}$
DE	$7.7042\times 10^{3}$	$5.5833\times 10^{3}$	$2.0480\times 10^{7}$	$1.1206\times 10^{8}$	$1.9943\times 10^{2}$	$1.5620\times 10^{2}$
CCO	$2.3807\times 10^{4}$	$1.0551\times 10^{4}$	$1.9650\times 10^{4}$	$1.1618\times 10^{4}$	$4.3222\times 10^{2}$	$2.0465\times 10^{2}$
GWO	$1.5001\times 10^{6}$	$4.8676\times 10^{5}$	$9.8798\times 10^{9}$	$3.3914\times 10^{9}$	$1.8075\times 10^{3}$	$2.7279\times 10^{2}$
PSO	$4.9126\times 10^{5}$	$2.0999\times 10^{6}$	$4.5611\times 10^{9}$	$1.4827\times 10^{10}$	$3.9482\times 10^{2}$	$2.7877\times 10^{2}$
CPO	$1.0738\times 10^{4}$	$6.4119\times 10^{3}$	$4.9036\times 10^{3}$	$8.5291\times 10^{3}$	$1.7273\times 10^{2}$	$1.3550\times 10^{2}$
ESO	$2.2437\times 10^{5}$	$9.2127\times 10^{4}$	$8.2603\times 10^{5}$	$2.3424\times 10^{5}$	$1.2160\times 10^{3}$	$6.2203\times 10^{2}$
SFO	$2.2589\times 10^{5}$	$1.2929\times 10^{5}$	$1.1641\times 10^{5}$	$5.6440\times 10^{4}$	$6.5981\times 10^{2}$	$4.0455\times 10^{2}$
SBO	$1.5690\times 10^{3}$	$2.3040\times 10^{3}$	$7.2123\times 10^{3}$	$1.2295\times 10^{4}$	$2.6905\times 10^{2}$	$1.4984\times 10^{2}$
	**F20**		**F21**		**F22**	
**Algo.**	**Avg**	**Std**	**Avg**	**Std**	**Avg**	**Std**
DCCO	$2.0261\times 10^{2}$	$9.4395\times 10^{1}$	$1.7876\times 10^{2}$	$1.1133\times 10^{1}$	$2.2978\times 10^{2}$	$6.8370\times 10^{1}$
DE	$3.1657\times 10^{2}$	$2.3424\times 10^{2}$	$1.9187\times 10^{2}$	$1.5551\times 10^{1}$	$9.5474\times 10^{2}$	$1.5996\times 10^{3}$
CCO	$3.6830\times 10^{3}$	$1.7261\times 10^{3}$	$4.0150\times 10^{2}$	$1.0413\times 10^{2}$	$1.4223\times 10^{4}$	$6.0702\times 10^{3}$
GWO	$4.6642\times 10^{3}$	$1.9993\times 10^{3}$	$2.8592\times 10^{2}$	$1.2436\times 10^{1}$	$1.1742\times 10^{4}$	$1.2929\times 10^{3}$
PSO	$2.9090\times 10^{3}$	$1.8781\times 10^{3}$	$3.2425\times 10^{2}$	$8.5279\times 10^{1}$	$1.1136\times 10^{4}$	$4.6284\times 10^{3}$
CPO	$1.5812\times 10^{2}$	$7.8109\times 10^{1}$	$1.6864\times 10^{2}$	$4.7951\times 10^{0}$	$2.1237\times 10^{2}$	$5.2496\times 10^{1}$
ESO	$4.7644\times 10^{2}$	$1.2314\times 10^{2}$	$1.8166\times 10^{2}$	$4.6132\times 10^{0}$	$1.1081\times 10^{3}$	$2.1670\times 10^{2}$
SFO	$1.9984\times 10^{2}$	$2.1073\times 10^{2}$	$2.7753\times 10^{2}$	$5.2285\times 10^{1}$	$2.5422\times 10^{2}$	$2.9698\times 10^{2}$
SBO	$2.5007\times 10^{2}$	$1.7845\times 10^{2}$	$1.6021\times 10^{2}$	$1.8009\times 10^{0}$	$2.5436\times 10^{2}$	$1.5604\times 10^{2}$
	**F23**		**F24**		**F25**	
**Algo.**	**Avg**	**Std**	**Avg**	**Std**	**Avg**	**Std**
DCCO	$2.1719\times 10^{2}$	$5.2551\times 10^{1}$	$4.2306\times 10^{2}$	$6.1571\times 10^{0}$	$8.2859\times 10^{2}$	$2.1461\times 10^{0}$
DE	$4.2512\times 10^{2}$	$4.1345\times 10^{2}$	$4.2622\times 10^{2}$	$7.7205\times 10^{0}$	$8.4515\times 10^{2}$	$2.0210\times 10^{1}$
CCO	$9.2623\times 10^{3}$	$2.7855\times 10^{3}$	$9.7447\times 10^{2}$	$2.3609\times 10^{2}$	$1.0873\times 10^{3}$	$1.8605\times 10^{2}$
GWO	$7.9525\times 10^{3}$	$6.4316\times 10^{2}$	$6.9647\times 10^{2}$	$3.6240\times 10^{1}$	$1.0198\times 10^{3}$	$6.1151\times 10^{1}$
PSO	$6.2220\times 10^{3}$	$3.5461\times 10^{3}$	$4.6425\times 10^{2}$	$5.3951\times 10^{1}$	$1.0841\times 10^{3}$	$1.7321\times 10^{2}$
CPO	$2.0975\times 10^{2}$	$3.7271\times 10^{1}$	$4.2263\times 10^{2}$	$4.9795\times 10^{0}$	$8.2893\times 10^{2}$	$9.1168\times 10^{0}$
ESO	$8.3479\times 10^{2}$	$8.5608\times 10^{1}$	$4.3079\times 10^{2}$	$2.0626\times 10^{1}$	$8.4034\times 10^{2}$	$1.6460\times 10^{1}$
SFO	$2.7020\times 10^{2}$	$2.6746\times 10^{2}$	$4.4454\times 10^{2}$	$3.5752\times 10^{1}$	$8.8824\times 10^{2}$	$2.5160\times 10^{1}$
SBO	$2.8481\times 10^{2}$	$1.9712\times 10^{2}$	$4.3201\times 10^{2}$	$1.2993\times 10^{1}$	$8.2842\times 10^{2}$	$2.0457\times 10^{0}$
	**F26**		**F27**		**F28**	
**Algo.**	**Avg**	**Std**	**Avg**	**Std**	**Avg**	**Std**
DCCO	$5.1681\times 10^{2}$	$1.3711\times 10^{1}$	$4.2647\times 10^{1}$	$7.4164\times 10^{1}$	$4.0906\times 10^{3}$	$2.1525\times 10^{3}$
DE	$5.2382\times 10^{2}$	$1.8211\times 10^{1}$	$9.0005\times 10^{1}$	$1.0526\times 10^{2}$	$5.2014\times 10^{3}$	$6.5665\times 10^{3}$
CCO	$6.1403\times 10^{2}$	$9.0804\times 10^{1}$	$6.7827\times 10^{2}$	$1.2581\times 10^{2}$	$1.5189\times 10^{5}$	$4.5197\times 10^{5}$
GWO	$6.2697\times 10^{2}$	$2.4706\times 10^{1}$	$6.5544\times 10^{2}$	$2.4838\times 10^{1}$	$9.0147\times 10^{8}$	$4.6551\times 10^{8}$
PSO	$6.3575\times 10^{2}$	$8.6879\times 10^{1}$	$8.4354\times 10^{2}$	$3.3865\times 10^{2}$	$5.1219\times 10^{7}$	$1.2014\times 10^{8}$
CPO	$5.1001\times 10^{2}$	$9.9107\times 10^{0}$	$2.8589\times 10^{-4}$	$2.4865\times 10^{-4}$	$2.9112\times 10^{3}$	$1.0563\times 10^{3}$
ESO	$5.5992\times 10^{2}$	$2.8942\times 10^{1}$	$1.0211\times 10^{2}$	$9.9991\times 10^{1}$	$3.1721\times 10^{4}$	$1.7940\times 10^{4}$
SFO	$5.4922\times 10^{2}$	$2.6645\times 10^{1}$	$3.6166\times 10^{2}$	$1.6629\times 10^{2}$	$8.0468\times 10^{4}$	$1.6297\times 10^{5}$
SBO	$5.4146\times 10^{2}$	$2.6712\times 10^{1}$	$3.8682\times 10^{1}$	$9.8116\times 10^{0}$	$3.5394\times 10^{3}$	$1.1180\times 10^{3}$
	**F29**		**F30**			
**Algo.**	**Avg**	**Std**	**Avg**	**Std**		
DCCO	$3.6831\times 10^{3}$	$3.5230\times 10^{3}$	$2.6000\times 10^{3}$	$8.9018\times 10^{2}$		
DE	$1.2725\times 10^{4}$	$1.1041\times 10^{4}$	$5.4401\times 10^{3}$	$2.3832\times 10^{3}$		
CCO	$2.9717\times 10^{5}$	$5.1566\times 10^{5}$	$7.1611\times 10^{4}$	$5.9803\times 10^{4}$		
GWO	$1.9860\times 10^{9}$	$6.3387\times 10^{8}$	$4.2555\times 10^{8}$	$6.9568\times 10^{7}$		
PSO	$5.6743\times 10^{8}$	$1.3552\times 10^{9}$	$1.5882\times 10^{6}$	$3.3944\times 10^{6}$		
CPO	$1.8831\times 10^{4}$	$1.6138\times 10^{4}$	$6.0521\times 10^{3}$	$3.5403\times 10^{3}$		
ESO	$2.1233\times 10^{6}$	$9.2312\times 10^{5}$	$1.1810\times 10^{7}$	$6.0943\times 10^{6}$		
SFO	$5.3669\times 10^{6}$	$1.4898\times 10^{7}$	$4.7292\times 10^{6}$	$8.3681\times 10^{6}$		
SBO	$8.9042\times 10^{3}$	$9.6050\times 10^{3}$	$4.5002\times 10^{3}$	$2.3803\times 10^{3}$		
**Overall Rank**						
**Algo.**	**RANK**	**+/=/−**	**AVG Rank**	**Avg Time (s)**		
DCCO	1	∼	1.7586	99.8104		
DE	4	27/0/2	3.6552	80.9845		
CCO	8	29/0/0	7.0000	75.7063		
GWO	9	29/0/0	8.2759	77.7431		
PSO	7	29/0/0	6.9310	73.1712		
CPO	2	17/0/12	2.2069	81.1157		
ESO	6	29/0/0	6.2414	76.9202		
SFO	5	28/0/1	5.8276	74.1580		
SBO	3	22/0/7	3.1034	68.9330		

**Table 3:** The *p*-values of the DCCO versus other algorithms on CEC2017.

Function	DE	CCO	GWO	PSO	CPO	ESO	SFO	SBO
F1	$2.51\times 10^{-9}$	$2.51\times 10^{-9}$	$2.51\times 10^{-9}$	$2.51\times 10^{-9}$	$2.51\times 10^{-9}$	$2.51\times 10^{-9}$	$2.51\times 10^{-9}$	$2.51\times 10^{-9}$
F3	$1.09\times 10^{-8}$	$2.78\times 10^{-9}$	$2.78\times 10^{-9}$	$2.78\times 10^{-9}$	$2.31\times 10^{-3}$	$2.78\times 10^{-9}$	$1.09\times 10^{-8}$	$6.83\times 10^{-3}$
F4	$4.59\times 10^{-1}$	$6.14\times 10^{-9}$	$6.14\times 10^{-9}$	$6.14\times 10^{-9}$	$7.83\times 10^{-1}$	$6.14\times 10^{-9}$	$6.14\times 10^{-9}$	$6.14\times 10^{-9}$
F5	$3.34\times 10^{-2}$	$6.14\times 10^{-9}$	$6.14\times 10^{-9}$	$1.02\times 10^{-2}$	$7.38\times 10^{-1}$	$6.14\times 10^{-9}$	$5.34\times 10^{-7}$	$5.91\times 10^{-3}$
F6	$1.00\times 10^{0}$	$6.14\times 10^{-9}$	$6.14\times 10^{-9}$	$6.14\times 10^{-9}$	$7.62\times 10^{-8}$	$6.14\times 10^{-9}$	$6.14\times 10^{-9}$	$6.14\times 10^{-9}$
F7	$9.37\times 10^{-5}$	$6.14\times 10^{-9}$	$6.14\times 10^{-9}$	$6.14\times 10^{-9}$	$7.08\times 10^{-9}$	$6.14\times 10^{-9}$	$6.14\times 10^{-9}$	$6.14\times 10^{-9}$
F8	$1.65\times 10^{-2}$	$6.14\times 10^{-9}$	$6.02\times 10^{-9}$	$1.14\times 10^{-5}$	$6.99\times 10^{-4}$	$6.14\times 10^{-9}$	$6.14\times 10^{-9}$	$2.05\times 10^{-8}$
F9	$1.66\times 10^{-4}$	$6.14\times 10^{-9}$	$6.14\times 10^{-9}$	$8.21\times 10^{-6}$	$2.35\times 10^{-4}$	$6.14\times 10^{-9}$	$6.14\times 10^{-9}$	$2.99\times 10^{-8}$
F10	$4.16\times 10^{-8}$	$6.14\times 10^{-9}$	$6.14\times 10^{-9}$	$6.14\times 10^{-9}$	$1.00\times 10^{0}$	$6.14\times 10^{-9}$	$6.54\times 10^{-9}$	$6.10\times 10^{-5}$
F11	$4.31\times 10^{-3}$	$6.14\times 10^{-9}$	$6.14\times 10^{-9}$	$6.14\times 10^{-9}$	$1.88\times 10^{-2}$	$6.14\times 10^{-9}$	$6.14\times 10^{-9}$	$1.00\times 10^{0}$
F12	$1.65\times 10^{-2}$	$6.14\times 10^{-9}$	$6.14\times 10^{-9}$	$6.14\times 10^{-9}$	$1.00\times 10^{0}$	$6.14\times 10^{-9}$	$6.14\times 10^{-9}$	$9.13\times 10^{-1}$
F13	$1.32\times 10^{-6}$	$3.54\times 10^{-8}$	$6.14\times 10^{-9}$	$2.74\times 10^{-8}$	$2.14\times 10^{-4}$	$6.14\times 10^{-9}$	$6.14\times 10^{-9}$	$1.76\times 10^{-4}$
F14	$2.81\times 10^{-7}$	$6.14\times 10^{-9}$	$6.14\times 10^{-9}$	$6.14\times 10^{-9}$	$1.77\times 10^{-8}$	$6.14\times 10^{-9}$	$6.14\times 10^{-9}$	$2.00\times 10^{-6}$
F15	$1.00\times 10^{0}$	$9.29\times 10^{-9}$	$6.14\times 10^{-9}$	$7.08\times 10^{-9}$	$6.25\times 10^{-2}$	$6.14\times 10^{-9}$	$6.14\times 10^{-9}$	$4.59\times 10^{-1}$
F16	$1.88\times 10^{-1}$	$3.86\times 10^{-8}$	$6.14\times 10^{-9}$	$7.41\times 10^{-7}$	$7.83\times 10^{-1}$	$4.16\times 10^{-8}$	$2.51\times 10^{-8}$	$1.00\times 10^{0}$
F17	$2.23\times 10^{-3}$	$6.39\times 10^{-8}$	$6.14\times 10^{-9}$	$4.92\times 10^{-8}$	$2.48\times 10^{-4}$	$6.14\times 10^{-9}$	$6.14\times 10^{-9}$	$5.11\times 10^{-1}$
F18	$4.92\times 10^{-8}$	$6.14\times 10^{-9}$	$6.14\times 10^{-9}$	$6.14\times 10^{-9}$	$8.27\times 10^{-8}$	$6.14\times 10^{-9}$	$6.14\times 10^{-9}$	$8.27\times 10^{-8}$
F19	$1.00\times 10^{0}$	$6.30\times 10^{-4}$	$6.14\times 10^{-9}$	$3.75\times 10^{-3}$	$1.00\times 10^{0}$	$2.99\times 10^{-8}$	$1.10\times 10^{-5}$	$3.11\times 10^{-2}$
F20	$1.21\times 10^{-1}$	$5.57\times 10^{-9}$	$5.57\times 10^{-9}$	$5.57\times 10^{-9}$	$1.00\times 10^{0}$	$2.46\times 10^{-8}$	$1.00\times 10^{0}$	$4.59\times 10^{-1}$
F21	$5.70\times 10^{-3}$	$6.14\times 10^{-9}$	$6.14\times 10^{-9}$	$6.14\times 10^{-9}$	$5.60\times 10^{-4}$	$3.75\times 10^{-1}$	$6.14\times 10^{-9}$	$6.03\times 10^{-9}$
F22	$2.41\times 10^{-6}$	$1.83\times 10^{-9}$	$1.83\times 10^{-9}$	$1.83\times 10^{-9}$	$1.25\times 10^{-3}$	$1.83\times 10^{-9}$	$2.14\times 10^{-4}$	$6.18\times 10^{-3}$
F23	$4.78\times 10^{-7}$	$2.78\times 10^{-9}$	$2.78\times 10^{-9}$	$2.78\times 10^{-9}$	$4.09\times 10^{-6}$	$2.78\times 10^{-9}$	$2.73\times 10^{-6}$	$9.56\times 10^{-6}$
F24	$1.84\times 10^{-2}$	$6.14\times 10^{-9}$	$6.14\times 10^{-9}$	$1.23\times 10^{-6}$	$1.00\times 10^{0}$	$4.09\times 10^{-6}$	$5.34\times 10^{-7}$	$1.10\times 10^{-5}$
F25	$2.19\times 10^{-7}$	$6.14\times 10^{-9}$	$6.14\times 10^{-9}$	$6.14\times 10^{-9}$	$1.88\times 10^{-3}$	$2.38\times 10^{-7}$	$6.54\times 10^{-9}$	$3.90\times 10^{-3}$
F26	$1.00\times 10^{0}$	$3.86\times 10^{-8}$	$6.14\times 10^{-9}$	$1.77\times 10^{-8}$	$8.42\times 10^{-1}$	$5.10\times 10^{-6}$	$2.15\times 10^{-5}$	$1.58\times 10^{-3}$
F27	$1.46\times 10^{-2}$	$5.57\times 10^{-9}$	$5.57\times 10^{-9}$	$5.57\times 10^{-9}$	$1.00\times 10^{0}$	$1.53\times 10^{-3}$	$3.81\times 10^{-7}$	$1.00\times 10^{-2}$
F28	$1.00\times 10^{0}$	$6.14\times 10^{-9}$	$6.14\times 10^{-9}$	$9.29\times 10^{-9}$	$9.83\times 10^{-1}$	$6.14\times 10^{-9}$	$2.81\times 10^{-7}$	$1.00\times 10^{0}$
F29	$2.15\times 10^{-5}$	$6.14\times 10^{-9}$	$6.14\times 10^{-9}$	$8.50\times 10^{-9}$	$3.56\times 10^{-7}$	$6.14\times 10^{-9}$	$6.14\times 10^{-9}$	$1.18\times 10^{-2}$
F30	$2.81\times 10^{-7}$	$6.14\times 10^{-9}$	$6.14\times 10^{-9}$	$6.14\times 10^{-9}$	$1.28\times 10^{-7}$	$6.14\times 10^{-9}$	$6.14\times 10^{-9}$	$2.22\times 10^{-4}$

**Table 4:** Ablation study results for DCCO on CEC 2017. “Best” signifies the number of functions where the variant outperformed others. “Mean Rank” denotes the average ranking. The “+/≈/−” column summarizes the statistical test results (α=0.05) relative to DCCO. The most favorable results are highlighted in grey shading.

Algorithm	Best	Mean Rank	+/≈/−
DCCO	18	1.5862	∼
CCO-I	4	2.9310	3/5/21
CCO-CC	2	2.6897	1/8/20
CCO	5	2.7931	6/2/21

**Table 5:** Parameter sensitivity analysis results for DCCO on CEC 2017 under Latin hypercube sampling.

Design	*K*	σ	*M*	Response
53	5	0.1466	80	5.3103
26	4	0.1151	104	10.2414
43	6	0.1395	85	10.8966
9	4	0.0652	95	11.1379
2	6	0.2129	71	14.4828
42	6	0.1756	58	14.8276
17	5	0.0932	113	15.5517
27	7	0.2260	57	20.0345
51	7	0.1240	81	20.0690
48	7	0.1635	88	20.3103

**Table 6:** Across-function post hoc analysis calculated from the original displayed D=30 means. Two-sided normal-approximation rank comparisons use 29 function blocks and nine algorithms; Holm adjustment covers the eight control comparisons. No original numerical entry is changed.

Comparator	Mean Rank	Rank Gap	Holm *p*
DE	3.6552	1.8966	0.0251
CCO	7.0000	5.2414	$2.2\times 10^{-12}$
GWO	8.2759	6.5172	$1.03\times 10^{-18}$
PSO	6.9310	5.1724	$3.83\times 10^{-12}$
CPO	2.2069	0.4483	0.533
ESO	6.2414	4.4828	$2.29\times 10^{-9}$
SFO	5.8276	4.0690	$6.14\times 10^{-8}$
SBO	3.1034	1.3448	0.123

**Table 7:** Higher-dimensional comparison and relative runtime. W/T/L compares mean errors with CCO.

*D*	Algorithm	Mean Rank	Time Ratio	W/T/L
50	DCCO	2.0000	1.165	26/0/3
50	CCO	3.3793	1.000	0/29/0
50	DBRCCO	2.4138	1.224	21/0/8
50	ALTCCO	2.2069	1.206	22/0/7
100	DCCO	2.1034	1.159	20/0/9
100	CCO	3.1379	1.000	0/29/0
100	DBRCCO	2.4483	1.222	20/0/9
100	ALTCCO	2.3103	1.201	22/0/7

**Table 8:** Independent-sample effect-size presentation for F3. Intervals are pointwise 95% percentile bootstrap intervals (2000 resamples); Holm correction covers all 174 added dimension–function–comparator tests, not just the displayed rows.

*D*	Comparator	$A_{\mathrm{lower}}$	95% CI	Holm *p*
50	CCO	0.972	[0.928, 1.000]	$5.21\times 10^{-8}$
50	DBRCCO	0.973	[0.931, 1.000]	$4.77\times 10^{-8}$
50	ALTCCO	0.777	[0.646, 0.896]	0.0191
100	CCO	0.964	[0.919, 0.994]	$9.88\times 10^{-8}$
100	DBRCCO	0.916	[0.840, 0.973]	$4.36\times 10^{-6}$
100	ALTCCO	0.732	[0.587, 0.852]	0.138

**Table 9:** Additional coefficient comparison. Each column of ranks is compared only within these six settings.

$q_{v}$	Mean Rank
0.00	5.2759
0.20	3.0690
0.40	2.5172
0.60	2.0690
0.80	3.2414
1.00	4.8276

**Table 10:** Experimental Results of All Algorithms on the Wastewater Operational Optimization Problem. The bold value indicates the best result.

Algorithm	Mean OCI	Std	Best	Worst
DCCO	$3.4849\times 10^{0}$	$2.5505\times 10^{-2}$	$3.4277\times 10^{0}$	$3.5463\times 10^{0}$
CCO	$4.6245\times 10^{0}$	$5.1966\times 10^{-1}$	$3.5085\times 10^{0}$	$5.8455\times 10^{0}$
CPO	$4.6694\times 10^{0}$	$5.1865\times 10^{-1}$	$3.6518\times 10^{0}$	$5.5236\times 10^{0}$
ESO	$4.7466\times 10^{0}$	$6.3310\times 10^{-1}$	$3.7257\times 10^{0}$	$6.2843\times 10^{0}$
SFO	$4.5043\times 10^{0}$	$4.6495\times 10^{-1}$	$3.6744\times 10^{0}$	$5.4872\times 10^{0}$
SBO	$4.6053\times 10^{0}$	$5.9791\times 10^{-1}$	$3.8040\times 10^{0}$	$6.2550\times 10^{0}$
DE	$4.7285\times 10^{0}$	$6.8839\times 10^{-1}$	$3.4990\times 10^{0}$	$6.0859\times 10^{0}$
PSO	$4.6986\times 10^{0}$	$4.0795\times 10^{-1}$	$3.7770\times 10^{0}$	$5.5442\times 10^{0}$
GWO	$4.6168\times 10^{0}$	$5.2096\times 10^{-1}$	$3.8323\times 10^{0}$	$6.1796\times 10^{0}$

**Table 11:** Component-wise application display.

Variant	Mean OCI	Std	Rain Feasible	Storm Feasible
DCCO	3.4986	0.0219	30/30	0/30
CCO-I	4.3014	0.3264	30/30	7/30
CCO-CC	3.8307	0.1905	30/30	0/30
CCO	4.5550	0.4634	30/30	16/30

**Table 12:** Exact analytical reference for the stated static model. These values are calculated from the model equations, not produced by an optimizer. The three other decisions are n=0.5, r=5000, and w=50.

Load	$d^{*}$	OCI	$C_{\mathrm{NH}}$	$C_{\mathrm{NO}}$	$C_{\mathrm{COD}}$
Dry (1.00)	1.045087	2.793765	4.5000	4.6000	30.921358
Rain (1.15)	1.340315	3.357155	4.5000	5.2900	33.713019
Storm (1.35)	1.780233	4.455382	4.5000	6.2100	37.487591

## Data Availability

Following acceptance of this article, the complete DCCO implementation and the experimental data supporting this study will be available from the corresponding author by email upon reasonable request.

## Appendix A. Notation and Reproducibility

**Table A1:** Notation used in the method and evaluation.

Symbol	Meaning
N,D,T	Population size, dimension and number of sweeps
$B,n_{\mathrm{FE}}$	Evaluation limit and consumed objective calls
$\Omega ,l_{j},u_{j}$	Feasible box and coordinate bounds
$x_{i},x_{i,j}$	Individual vector and its coordinate
$x_{\mathrm{best}},\bar{x}$	Best evaluated point and population centroid
${\tilde{x}}_{i,j}$	Coordinate normalized to the unit interval
K,M,L	Cluster count, clustering sample count and clustering iterations
$z_{k},S_{k}$	Cluster center and assigned sample group
$\mu ,\sigma ,\xi_{i,j}$	Cauchy location, scale and perturbation
z,g,η	Baseline binary switch, standard normal draw and unit-uniform draw
$r_{1},\ldots ,r_{4}$	Sampled individual indices in the baseline compressed-space update
$C^{\left(t\right)},T_{s}^{\left(t\right)},w^{\left(t\right)}$	Baseline iteration-dependent factors; $w^{\left(t\right)}$ is distinct from the wastewater decision *w*
$y_{i},x_{i}^{+}$	Baseline trial vector and retained next vector
π,ρ	Random permutations of individuals and dimensions
$r_{1,j},r_{2,j},r_{3,i,b}$	Fresh uniform crossover coefficients on [0,1]
$c_{1,j},c_{2,j}$	Fresh crossover coefficients on [−1,1]
$p_{v},q_{v}$	Stage-level uniform draw and VCS activation probability
$H_{i,j},v_{i,j}$	Horizontal and vertical trial coordinates
B	Boundary-repair operator
$E_{A},A_{\mathrm{lower}}$	Error sample for method *A* and oriented effect size
d,n,r,w,l	Oxygen/nitrate setpoints, sludge flows and load multiplier
OCI, EQI	Operating cost index and effluent quality index

The shared main-comparison settings are D=30, N=30, 30 runs and B=300,000. Table A2 specifies a proposed replication configuration based on the cited algorithm definitions and explicit implementation choices. It lists fixed coefficients, population rules and adaptive schedules for all nine methods. Each method retains its native initialization structure, and every objective call, including initialization, is charged to the common evaluation budget. The parameter-sensitivity study evaluates separate configurations.

**Table A2:** Proposed replication configuration for DCCO and the eight comparison methods. Fixed values and adaptive rules are specified together; algorithm references identify the underlying methods.

Method and Source	Parameter Values and Update Rules
DCCO (this study)	K=5, M=80, μ=0, σ=0.1466 in original coordinates; $N_{c}=15$; VCS probability $q_{v}=0.6$. Lloyd clustering: one start, at most 100 iterations, center-displacement tolerance $10^{-6}$ after range normalization; initial centers sampled without replacement; empty centers retained. CCO controls as below.
CCO [19]	Spiral constants a=1.34, b=0.3; encirclement exponent 2, predation exponent 5, Lévy index 1.5. Native time-dependent stage factors; death coefficient 0.02. After 20 consecutive unsuccessful updates, reset C=0.8 and die=0.02.
CPO [46]	α=0.2, $T_{f}=0.8$; two population-reduction cycles; initial population $N_{0}=30$, minimum population $N_{\min}=24$; cyclic schedule retained.
ESO [47]	Adaptive resistance, conductivity and intensity follow the author’s Python (version 3.10) implementation; range-denominator floor $10^{-6}$, numerical offset $10^{-49}$; restart after more than two stagnant steps. No additional fixed search coefficient.
SFO [48]	w=0.5, $c_{1}=c_{2}=1.5$, exploration scale α=0.5, Lévy amplitude β=0.57, stable index 1.5; micro-search step $\epsilon =10^{-3}$. Failure thresholds: glide >4, target >10; micro phase after cycle 30; restart after stagnation >20, at most five resets.
SBO [49]	30 families, two members per family; $w_{1},w_{2}∼N\left(0,1\right)$; adaptive $w_{3}$ from Equation (5) of the source and $w_{4}∼U\left(-w_{3},w_{3}\right)$; roulette-wheel elite selection. All 60 initial evaluations count toward *B*.
DE [13]	DE/rand/1/bin; differential weight F=0.5; crossover probability CR=0.9; one forced donor coordinate; distinct donor indices.
PSO [14,48]	Global-best topology; w=0.5, $c_{1}=c_{2}=1.5$; $v_{\max,j}=0.2\left(u_{j}-l_{j}\right)$; initial velocities zero; velocity clipping followed by position clipping.
GWO [15]	*a* decreases linearly from 2 to 0 over the prescribed schedule; $A=2ar_{1}-a$, $C=2r_{2}$, with independent $r_{1},r_{2}∼U\left(0,1\right)$; three leading wolves.

## Appendix B. Additional Function-Level Results

Table A3 and Table A4 retain all 29 function entries. Bold values identify the smallest full-precision mean within each function and dimension; standard deviations use the sample denominator n−1.

**Table A3:** Additional comparison at D=50. All 29 functions and 30 runs per method are included. Bold values identify the smallest mean within each function.

	F1		F3		F4	
Algorithm	Avg	Std	Avg	Std	Avg	Std
DCCO	$4.3832\times 10^{10}$	$1.9725\times 10^{11}$	$7.1802\times 10^{2}$	$5.1628\times 10^{2}$	$2.0160\times 10^{4}$	$7.1123\times 10^{3}$
CCO	$3.0442\times 10^{10}$	$1.2559\times 10^{10}$	$2.9904\times 10^{3}$	$1.6809\times 10^{3}$	$4.4782\times 10^{4}$	$1.7340\times 10^{4}$
DBRCCO	$5.3215\times 10^{10}$	$2.1954\times 10^{10}$	$3.2443\times 10^{3}$	$1.8236\times 10^{3}$	$1.3862\times 10^{4}$	$5.3676\times 10^{3}$
ALTCCO	$2.3000\times 10^{10}$	$9.4886\times 10^{9}$	$1.1813\times 10^{3}$	$6.6399\times 10^{2}$	$7.5757\times 10^{3}$	$2.9334\times 10^{3}$
	F5		F6		F7	
Algorithm	Avg	Std	Avg	Std	Avg	Std
DCCO	$1.7800\times 10^{-3}$	$8.0101\times 10^{-3}$	$1.4718\times 10^{6}$	$3.0566\times 10^{5}$	$4.5693\times 10^{2}$	$8.1233\times 10^{1}$
CCO	$3.8192\times 10^{-3}$	$2.5366\times 10^{-3}$	$1.7609\times 10^{6}$	$6.0782\times 10^{5}$	$4.9150\times 10^{2}$	$8.3590\times 10^{1}$
DBRCCO	$1.9102\times 10^{-3}$	$1.2687\times 10^{-3}$	$1.1363\times 10^{6}$	$3.9223\times 10^{5}$	$3.9339\times 10^{2}$	$6.6903\times 10^{1}$
ALTCCO	$1.0521\times 10^{-3}$	$6.9874\times 10^{-4}$	$1.2179\times 10^{6}$	$4.2040\times 10^{5}$	$5.7340\times 10^{2}$	$9.7517\times 10^{1}$
	F8		F9		F10	
Algorithm	Avg	Std	Avg	Std	Avg	Std
DCCO	$5.0124\times 10^{0}$	$6.6332\times 10^{0}$	$5.9558\times 10^{3}$	$1.3211\times 10^{3}$	$7.3641\times 10^{4}$	$8.7855\times 10^{4}$
CCO	$1.5544\times 10^{1}$	$4.8369\times 10^{0}$	$1.2563\times 10^{4}$	$2.0172\times 10^{3}$	$1.3786\times 10^{5}$	$5.1911\times 10^{4}$
DBRCCO	$1.2587\times 10^{1}$	$3.9168\times 10^{0}$	$6.6906\times 10^{3}$	$1.0743\times 10^{3}$	$3.0658\times 10^{4}$	$1.1544\times 10^{4}$
ALTCCO	$1.7541\times 10^{1}$	$5.4583\times 10^{0}$	$2.0715\times 10^{4}$	$3.3262\times 10^{3}$	$4.3844\times 10^{4}$	$1.6510\times 10^{4}$
	F11		F12		F13	
Algorithm	Avg	Std	Avg	Std	Avg	Std
DCCO	$1.7020\times 10^{7}$	$1.6292\times 10^{7}$	$4.3888\times 10^{6}$	$4.3542\times 10^{6}$	$2.7249\times 10^{4}$	$5.8893\times 10^{4}$
CCO	$2.3995\times 10^{7}$	$2.7158\times 10^{7}$	$7.5489\times 10^{6}$	$1.5276\times 10^{7}$	$4.5204\times 10^{4}$	$3.2460\times 10^{4}$
DBRCCO	$5.2737\times 10^{7}$	$5.9688\times 10^{7}$	$3.4182\times 10^{6}$	$6.9171\times 10^{6}$	$2.1933\times 10^{4}$	$1.5750\times 10^{4}$
ALTCCO	$1.6230\times 10^{7}$	$1.8370\times 10^{7}$	$2.4389\times 10^{6}$	$4.9354\times 10^{6}$	$3.9705\times 10^{4}$	$2.8511\times 10^{4}$
	F14		F15		F16	
Algorithm	Avg	Std	Avg	Std	Avg	Std
DCCO	$1.7489\times 10^{5}$	$2.4069\times 10^{5}$	$1.1300\times 10^{3}$	$7.7446\times 10^{2}$	$9.1840\times 10^{3}$	$9.1295\times 10^{3}$
CCO	$3.9395\times 10^{5}$	$8.6045\times 10^{5}$	$6.5447\times 10^{3}$	$5.3204\times 10^{3}$	$1.3468\times 10^{4}$	$2.4393\times 10^{4}$
DBRCCO	$2.7847\times 10^{5}$	$6.0822\times 10^{5}$	$1.8965\times 10^{3}$	$1.5418\times 10^{3}$	$1.1089\times 10^{4}$	$2.0084\times 10^{4}$
ALTCCO	$3.2459\times 10^{5}$	$7.0896\times 10^{5}$	$1.1368\times 10^{3}$	$9.2417\times 10^{2}$	$8.6438\times 10^{3}$	$1.5656\times 10^{4}$
	F17		F18		F19	
Algorithm	Avg	Std	Avg	Std	Avg	Std
DCCO	$2.4507\times 10^{4}$	$4.5506\times 10^{4}$	$2.9206\times 10^{4}$	$5.4439\times 10^{4}$	$5.4359\times 10^{2}$	$5.0441\times 10^{2}$
CCO	$5.1225\times 10^{4}$	$2.2702\times 10^{4}$	$4.2280\times 10^{4}$	$2.4998\times 10^{4}$	$9.2999\times 10^{2}$	$4.4034\times 10^{2}$
DBRCCO	$3.8889\times 10^{4}$	$1.7235\times 10^{4}$	$2.7277\times 10^{4}$	$1.6127\times 10^{4}$	$9.3454\times 10^{2}$	$4.4249\times 10^{2}$
ALTCCO	$2.2269\times 10^{4}$	$9.8694\times 10^{3}$	$6.0793\times 10^{4}$	$3.5944\times 10^{4}$	$6.0863\times 10^{2}$	$2.8818\times 10^{2}$
	F20		F21		F22	
Algorithm	Avg	Std	Avg	Std	Avg	Std
DCCO	$6.9835\times 10^{3}$	$3.2536\times 10^{3}$	$6.0245\times 10^{2}$	$3.7520\times 10^{1}$	$1.5674\times 10^{4}$	$4.6636\times 10^{3}$
CCO	$7.9246\times 10^{3}$	$3.7140\times 10^{3}$	$8.6389\times 10^{2}$	$2.2405\times 10^{2}$	$3.0603\times 10^{4}$	$1.3061\times 10^{4}$
DBRCCO	$3.3082\times 10^{3}$	$1.5504\times 10^{3}$	$4.6836\times 10^{2}$	$1.2147\times 10^{2}$	$2.5005\times 10^{4}$	$1.0672\times 10^{4}$
ALTCCO	$4.9283\times 10^{3}$	$2.3097\times 10^{3}$	$1.1769\times 10^{3}$	$3.0524\times 10^{2}$	$1.2114\times 10^{4}$	$5.1703\times 10^{3}$
	F23		F24		F25	
Algorithm	Avg	Std	Avg	Std	Avg	Std
DCCO	$5.7850\times 10^{3}$	$1.3997\times 10^{3}$	$8.5977\times 10^{2}$	$1.2513\times 10^{1}$	$6.1702\times 10^{3}$	$3.0851\times 10^{1}$
CCO	$1.9929\times 10^{4}$	$5.9934\times 10^{3}$	$2.0967\times 10^{3}$	$5.0798\times 10^{2}$	$2.3395\times 10^{3}$	$4.0032\times 10^{2}$
DBRCCO	$1.1886\times 10^{4}$	$3.5744\times 10^{3}$	$1.0126\times 10^{3}$	$2.4533\times 10^{2}$	$5.3011\times 10^{3}$	$9.0708\times 10^{2}$
ALTCCO	$7.9432\times 10^{3}$	$2.3888\times 10^{3}$	$8.9931\times 10^{2}$	$2.1788\times 10^{2}$	$2.2176\times 10^{3}$	$3.7946\times 10^{2}$
	F26		F27		F28	
Algorithm	Avg	Std	Avg	Std	Avg	Std
DCCO	$1.6296\times 10^{2}$	$4.3235\times 10^{0}$	$6.8569\times 10^{2}$	$1.1924\times 10^{3}$	$1.5364\times 10^{5}$	$8.0844\times 10^{4}$
CCO	$1.3212\times 10^{3}$	$1.9538\times 10^{2}$	$1.4594\times 10^{3}$	$2.7070\times 10^{2}$	$3.2682\times 10^{5}$	$9.7248\times 10^{5}$
DBRCCO	$2.4596\times 10^{2}$	$3.6373\times 10^{1}$	$1.9434\times 10^{3}$	$3.6047\times 10^{2}$	$1.3001\times 10^{5}$	$3.8687\times 10^{5}$
ALTCCO	$3.4531\times 10^{2}$	$5.1066\times 10^{1}$	$6.7129\times 10^{2}$	$1.2452\times 10^{2}$	$1.7877\times 10^{5}$	$5.3196\times 10^{5}$
	F29		F30			
Algorithm	Avg	Std	Avg	Std		
DCCO	$1.1616\times 10^{6}$	$1.1112\times 10^{6}$	$1.1670\times 10^{5}$	$3.9956\times 10^{4}$		
CCO	$6.3941\times 10^{5}$	$1.1095\times 10^{6}$	$1.5408\times 10^{5}$	$1.2868\times 10^{5}$		
DBRCCO	$7.7337\times 10^{5}$	$1.3420\times 10^{6}$	$3.9776\times 10^{5}$	$3.3217\times 10^{5}$		
ALTCCO	$8.2623\times 10^{5}$	$1.4337\times 10^{6}$	$1.8163\times 10^{5}$	$1.5168\times 10^{5}$		

**Table A4:** Additional comparison at D=100. All 29 functions and 30 runs per method are included. Bold values identify the smallest mean within each function.

	F1		F3		F4	
Algorithm	Avg	Std	Avg	Std	Avg	Std
DCCO	$1.4621\times 10^{11}$	$6.5793\times 10^{11}$	$1.7889\times 10^{3}$	$1.2863\times 10^{3}$	$1.3710\times 10^{5}$	$4.8369\times 10^{4}$
CCO	$8.6102\times 10^{10}$	$3.5521\times 10^{10}$	$8.4581\times 10^{3}$	$4.7542\times 10^{3}$	$1.2666\times 10^{5}$	$4.9046\times 10^{4}$
DBRCCO	$8.0839\times 10^{10}$	$3.3350\times 10^{10}$	$5.4206\times 10^{3}$	$3.0469\times 10^{3}$	$3.0744\times 10^{5}$	$1.1904\times 10^{5}$
ALTCCO	$1.0991\times 10^{11}$	$4.5343\times 10^{10}$	$2.9633\times 10^{3}$	$1.6657\times 10^{3}$	$2.2851\times 10^{5}$	$8.8483\times 10^{4}$
	F5		F6		F7	
Algorithm	Avg	Std	Avg	Std	Avg	Std
DCCO	$1.2945\times 10^{-2}$	$5.8253\times 10^{-2}$	$9.7938\times 10^{5}$	$2.0339\times 10^{5}$	$6.0238\times 10^{2}$	$1.0709\times 10^{2}$
CCO	$1.0802\times 10^{-2}$	$7.1746\times 10^{-3}$	$4.9804\times 10^{6}$	$1.7192\times 10^{6}$	$1.3902\times 10^{3}$	$2.3643\times 10^{2}$
DBRCCO	$1.0275\times 10^{-2}$	$6.8241\times 10^{-3}$	$1.0080\times 10^{6}$	$3.4796\times 10^{5}$	$1.7101\times 10^{3}$	$2.9083\times 10^{2}$
ALTCCO	$1.3356\times 10^{-2}$	$8.8704\times 10^{-3}$	$3.3304\times 10^{6}$	$1.1496\times 10^{6}$	$9.4175\times 10^{2}$	$1.6016\times 10^{2}$
	F8		F9		F10	
Algorithm	Avg	Std	Avg	Std	Avg	Std
DCCO	$5.7792\times 10^{1}$	$7.6480\times 10^{1}$	$1.1538\times 10^{4}$	$2.5593\times 10^{3}$	$1.8159\times 10^{5}$	$2.1664\times 10^{5}$
CCO	$4.3966\times 10^{1}$	$1.3681\times 10^{1}$	$3.5533\times 10^{4}$	$5.7054\times 10^{3}$	$3.8992\times 10^{5}$	$1.4683\times 10^{5}$
DBRCCO	$4.6613\times 10^{1}$	$1.4505\times 10^{1}$	$9.3040\times 10^{3}$	$1.4939\times 10^{3}$	$3.6627\times 10^{5}$	$1.3792\times 10^{5}$
ALTCCO	$7.4596\times 10^{1}$	$2.3212\times 10^{1}$	$1.6908\times 10^{4}$	$2.7149\times 10^{3}$	$1.1763\times 10^{5}$	$4.4295\times 10^{4}$
	F11		F12		F13	
Algorithm	Avg	Std	Avg	Std	Avg	Std
DCCO	$4.5711\times 10^{7}$	$4.3758\times 10^{7}$	$7.9540\times 10^{6}$	$7.8913\times 10^{6}$	$6.5046\times 10^{4}$	$1.4058\times 10^{5}$
CCO	$6.7869\times 10^{7}$	$7.6815\times 10^{7}$	$2.1351\times 10^{7}$	$4.3207\times 10^{7}$	$1.2786\times 10^{5}$	$9.1810\times 10^{4}$
DBRCCO	$2.5329\times 10^{7}$	$2.8668\times 10^{7}$	$8.7075\times 10^{6}$	$1.7621\times 10^{7}$	$1.1809\times 10^{5}$	$8.4799\times 10^{4}$
ALTCCO	$6.3248\times 10^{7}$	$7.1585\times 10^{7}$	$4.8742\times 10^{6}$	$9.8635\times 10^{6}$	$8.1983\times 10^{4}$	$5.8869\times 10^{4}$
	F14		F15		F16	
Algorithm	Avg	Std	Avg	Std	Avg	Std
DCCO	$3.0553\times 10^{5}$	$4.2047\times 10^{5}$	$1.2444\times 10^{4}$	$8.5287\times 10^{3}$	$1.9310\times 10^{4}$	$1.9195\times 10^{4}$
CCO	$1.1143\times 10^{6}$	$2.4337\times 10^{6}$	$1.8511\times 10^{4}$	$1.5048\times 10^{4}$	$3.8092\times 10^{4}$	$6.8995\times 10^{4}$
DBRCCO	$6.5249\times 10^{5}$	$1.4251\times 10^{6}$	$7.9678\times 10^{3}$	$6.4773\times 10^{3}$	$6.5157\times 10^{4}$	$1.1802\times 10^{5}$
ALTCCO	$5.5927\times 10^{5}$	$1.2215\times 10^{6}$	$9.0090\times 10^{3}$	$7.3237\times 10^{3}$	$2.4728\times 10^{4}$	$4.4790\times 10^{4}$
	F17		F18		F19	
Algorithm	Avg	Std	Avg	Std	Avg	Std
DCCO	$4.5115\times 10^{4}$	$8.3773\times 10^{4}$	$9.2850\times 10^{4}$	$1.7307\times 10^{5}$	$1.4205\times 10^{3}$	$1.3181\times 10^{3}$
CCO	$1.4488\times 10^{5}$	$6.4211\times 10^{4}$	$1.1959\times 10^{5}$	$7.0705\times 10^{4}$	$2.6304\times 10^{3}$	$1.2455\times 10^{3}$
DBRCCO	$1.6663\times 10^{5}$	$7.3849\times 10^{4}$	$9.7239\times 10^{4}$	$5.7492\times 10^{4}$	$1.1176\times 10^{3}$	$5.2916\times 10^{2}$
ALTCCO	$1.0173\times 10^{5}$	$4.5087\times 10^{4}$	$4.3659\times 10^{4}$	$2.5813\times 10^{4}$	$1.6065\times 10^{3}$	$7.6067\times 10^{2}$
	F20		F21		F22	
Algorithm	Avg	Std	Avg	Std	Avg	Std
DCCO	$2.4644\times 10^{4}$	$1.1482\times 10^{4}$	$6.3341\times 10^{2}$	$3.9448\times 10^{1}$	$2.9115\times 10^{4}$	$8.6630\times 10^{3}$
CCO	$2.2414\times 10^{4}$	$1.0505\times 10^{4}$	$2.4435\times 10^{3}$	$6.3371\times 10^{2}$	$8.6558\times 10^{4}$	$3.6942\times 10^{4}$
DBRCCO	$3.6407\times 10^{4}$	$1.7063\times 10^{4}$	$1.4621\times 10^{3}$	$3.7919\times 10^{2}$	$6.2909\times 10^{4}$	$2.6849\times 10^{4}$
ALTCCO	$2.5753\times 10^{4}$	$1.2069\times 10^{4}$	$5.7398\times 10^{2}$	$1.4886\times 10^{2}$	$3.9027\times 10^{4}$	$1.6656\times 10^{4}$
	F23		F24		F25	
Algorithm	Avg	Std	Avg	Std	Avg	Std
DCCO	$5.1754\times 10^{4}$	$1.2522\times 10^{4}$	$3.9401\times 10^{3}$	$5.7343\times 10^{1}$	$1.6664\times 10^{4}$	$8.3319\times 10^{1}$
CCO	$5.6369\times 10^{4}$	$1.6952\times 10^{4}$	$5.9304\times 10^{3}$	$1.4368\times 10^{3}$	$6.6171\times 10^{3}$	$1.1323\times 10^{3}$
DBRCCO	$4.5039\times 10^{4}$	$1.3545\times 10^{4}$	$2.4021\times 10^{3}$	$5.8198\times 10^{2}$	$7.9990\times 10^{3}$	$1.3687\times 10^{3}$
ALTCCO	$4.3235\times 10^{4}$	$1.3002\times 10^{4}$	$4.7946\times 10^{3}$	$1.1616\times 10^{3}$	$5.2740\times 10^{3}$	$9.0245\times 10^{2}$
	F26		F27		F28	
Algorithm	Avg	Std	Avg	Std	Avg	Std
DCCO	$4.0189\times 10^{3}$	$1.0662\times 10^{2}$	$3.1685\times 10^{3}$	$5.5101\times 10^{3}$	$1.1185\times 10^{6}$	$5.8857\times 10^{5}$
CCO	$3.7369\times 10^{3}$	$5.5262\times 10^{2}$	$4.1278\times 10^{3}$	$7.6566\times 10^{2}$	$9.2437\times 10^{5}$	$2.7506\times 10^{6}$
DBRCCO	$2.2288\times 10^{3}$	$3.2960\times 10^{2}$	$5.0647\times 10^{3}$	$9.3943\times 10^{2}$	$1.0821\times 10^{6}$	$3.2199\times 10^{6}$
ALTCCO	$5.2449\times 10^{3}$	$7.7562\times 10^{2}$	$3.9987\times 10^{3}$	$7.4170\times 10^{2}$	$1.3386\times 10^{6}$	$3.9833\times 10^{6}$
	F29		F30			
Algorithm	Avg	Std	Avg	Std		
DCCO	$1.8638\times 10^{6}$	$1.7828\times 10^{6}$	$2.1853\times 10^{5}$	$7.4819\times 10^{4}$		
CCO	$1.8085\times 10^{6}$	$3.1382\times 10^{6}$	$4.3581\times 10^{5}$	$3.6395\times 10^{5}$		
DBRCCO	$9.2904\times 10^{5}$	$1.6121\times 10^{6}$	$3.4271\times 10^{5}$	$2.8620\times 10^{5}$		
ALTCCO	$1.3784\times 10^{6}$	$2.3918\times 10^{6}$	$1.1315\times 10^{5}$	$9.4497\times 10^{4}$		

## Appendix C. Complete Parameter Display

**Table A5:** Complete 60-configuration display.

Design	*K*	σ	*M*	Response
1	7	0.0673	44	40.3448
2	8	0.2309	51	55.7931
3	8	0.1734	91	43.8276
4	4	0.2399	80	25.2414
5	5	0.0884	77	4.6552
6	4	0.1044	125	16.0345
7	4	0.2342	120	31.2759
8	7	0.2101	106	35.8966
9	3	0.1881	123	31.1379
10	6	0.0832	94	8.4483
11	3	0.1944	85	19.9655
12	3	0.1634	138	41.1034
13	5	0.0324	130	45.1379
14	4	0.1360	60	9.1724
15	6	0.0959	117	18.0345
16	7	0.1387	124	38.1724
17	5	0.2546	132	51.0690
18	3	0.0560	49	49.1379
19	7	0.1235	83	13.2069
20	6	0.2608	63	41.0345
21	3	0.0794	134	43.7586
22	4	0.1681	136	22.7931
23	7	0.1459	80	12.3793
24	3	0.2015	99	22.6207
25	6	0.2735	114	51.3448
26	3	0.1834	116	23.5517
27	6	0.0614	53	24.3793
28	5	0.1175	75	3.2069
29	5	0.2663	74	34.6552
30	6	0.1577	58	9.0690
31	3	0.2688	72	53.1034
32	2	0.2005	139	57.1724
33	5	0.0708	128	27.3103
34	2	0.0858	93	43.2414
35	4	0.2238	85	16.3103
36	6	0.1004	96	7.0000
37	7	0.2585	41	55.1379
38	5	0.2497	46	41.4138
39	6	0.2450	102	30.1724
40	4	0.1549	47	18.3103
41	4	0.0391	121	36.1034
42	3	0.1160	87	13.3103
43	7	0.1796	43	30.4828
44	8	0.2765	131	60.0000
45	7	0.1105	109	24.3793
46	4	0.0375	103	27.4828
47	8	0.2061	90	49.4828
48	2	0.1485	107	41.8276
49	7	0.1910	112	32.1724
50	2	0.1325	59	49.5172
51	4	0.2266	55	29.6897
52	5	0.1592	70	3.4483
53	6	0.2172	98	15.3103
54	2	0.2176	55	57.3793
55	8	0.0495	66	54.1724
56	3	0.1091	64	23.1034
57	5	0.1273	111	4.2069
58	6	0.0444	67	21.7931
59	7	0.0752	70	25.1379
60	5	0.0538	100	15.3793
